# The Constrained Maximal Expression Level Owing to Haploidy Shapes Gene Content on the Mammalian X Chromosome

**DOI:** 10.1371/journal.pbio.1002315

**Published:** 2015-12-18

**Authors:** Laurence D. Hurst, Avazeh T. Ghanbarian, Alistair R. R. Forrest, FANTOM consortium, Lukasz Huminiecki

**Affiliations:** 1 The Milner Centre for Evolution, Department of Biology and Biochemistry, University of Bath, Bath, United Kingdom; 2 Center for Life Science Technologies (CLST), Yokohama, Japan; 3 The Department of Biochemistry and Biophysics, Stockholm University, Stockholm, Sweden; 4 Science for Life Laboratory, Stockholm, Sweden; 5 The Department of Cell and Molecular Biology, Karolinska Institutet, Stockholm, Sweden; 6 Bioinformatics Infrastructure for Life Sciences (BILS), Stockholm, Sweden; Institute of Science and Technology Austria (IST Austria), AUSTRIA

## Abstract

X chromosomes are unusual in many regards, not least of which is their nonrandom gene content. The causes of this bias are commonly discussed in the context of sexual antagonism and the avoidance of activity in the male germline. Here, we examine the notion that, at least in some taxa, functionally biased gene content may more profoundly be shaped by limits imposed on gene expression owing to haploid expression of the X chromosome. Notably, if the X, as in primates, is transcribed at rates comparable to the ancestral rate (per promoter) prior to the X chromosome formation, then the X is not a tolerable environment for genes with very high maximal net levels of expression, owing to transcriptional traffic jams. We test this hypothesis using The Encyclopedia of DNA Elements (ENCODE) and data from the Functional Annotation of the Mammalian Genome (FANTOM5) project. As predicted, the maximal expression of human X-linked genes is much lower than that of genes on autosomes: on average, maximal expression is three times lower on the X chromosome than on autosomes. Similarly, autosome-to-X retroposition events are associated with lower maximal expression of retrogenes on the X than seen for X-to-autosome retrogenes on autosomes. Also as expected, X-linked genes have a lesser degree of increase in gene expression than autosomal ones (compared to the human/Chimpanzee common ancestor) if highly expressed, but not if lowly expressed. The traffic jam model also explains the known lower breadth of expression for genes on the X (and the Z of birds), as genes with broad expression are, on average, those with high maximal expression. As then further predicted, highly expressed tissue-specific genes are also rare on the X and broadly expressed genes on the X tend to be lowly expressed, both indicating that the trend is shaped by the maximal expression level not the breadth of expression per se. Importantly, a limit to the maximal expression level explains biased tissue of expression profiles of X-linked genes. Tissues whose tissue-specific genes are very highly expressed (e.g., secretory tissues, tissues abundant in structural proteins) are also tissues in which gene expression is relatively rare on the X chromosome. These trends cannot be fully accounted for in terms of alternative models of biased expression. In conclusion, the notion that it is hard for genes on the Therian X to be highly expressed, owing to transcriptional traffic jams, provides a simple yet robustly supported rationale of many peculiar features of X’s gene content, gene expression, and evolution.

## Abstract 2

Laurence Hurst, Lukasz Huminiecki, and the FANTOM5 consortium propose a new explanation for the peculiar expression properties of genes on the human X chromosome, based on the premise that very high expression levels cannot be achieved on a haploid-expressed chromosome.

## Author Summary

Genes located on the human X chromosome are not a random mix of genes: they tend to be expressed in relatively few tissues or are specific for a particular set of tissues, e.g., brain regions. Prior attempts to explain this skewed gene content have hypothesized that the X chromosome might be peculiar because it has to balance mutations that are advantageous to one sex but deleterious to the other, or because it has to shut down during the process of sperm manufacture in males. Here we suggest and test a third possible explanation: that genes on the X chromosome are limited in their transcription levels and thus tend to be genes that are lowly or specifically expressed. We consider the suggestion that since these genes can only be expressed from one chromosome, as males only have one X, the ability to express a gene at very high rates is limited owing to potential transcriptional traffic jams. As predicted, we find that human X-located genes have maximal expression rates far below that of genes residing on autosomes. When we look at genes that have moved onto or off the X chromosome during recent evolution, we find the maximal expression is higher when not on the X chromosome. We also find that X-located genes that are relatively highly expressed are not able to increase their expression level further. Our model explains both the enrichment for tissue specificity and the paucity of certain tissues with X-located genes. Genes underrepresented on the X are either expressed in many tissues—such genes tend to have high maximal expression—or are from tissues that require a lot of transcription (e.g., fast secreting tissues like the liver). Just as many of the findings cannot be explained by the two earlier models, neither can the traffic jam model explain all the peculiar features of the genes found on the X chromosome. Indeed, we find evidence of a reproduction-related bias in X-located genes, even after allowing for the traffic jam problem.

## Introduction

X chromosomes are in many regards unusual (reviewed in [1,2]). The peculiarities of the X include an unusual recombination environment, unusual dominance relations and an unusual proportion of time spent in members of the two sexes. The consequences of these peculiarities include, in some taxa, reduced recombination rates and lower mutation rates. In addition, the X chromosome is expected to have an unusual effective population size, this being exaggerated by strong sexual selection [3–8]. Such forces can likely explain many peculiarities of X chromosomes. For example, the human X chromosome, whose genome sequence [9] is much less degraded than that of non-recombining Y chromosome, has an AT-content between that of the Y chromosome and autosomes, consistent with reduced rates of recombination-associated biased gene conversion favoring AT→GC SNPs in the face of a GC→AT mutation bias [10]. That we see an increase in GC-content as we move from the haploid part of the X to pseudoautosomal regions on the X [11,12] strongly supports such a recombination-associated model. The hemizygous nature of the X in males, exposing adaptive mutations, is similarly central to the “faster X” hypothesis—that is, the notion that X-linked genes might be fast evolving [5,6,13].

Several models also predict that the X will be unusual in its gene content. Two models are pre-eminent. First, Rice noted that because of dominance effects and time spent in the two sexes, an X chromosome might have a tendency to accumulate genes with either male- or female-specific expression [14]. The former is owing to a selective filter enabling the spread of male-advantage/female-disadvantage recessive alleles (the deleterious effects being hidden at the point of invasion of a sexually antagonistic allele). The latter is owing to selection favoring dominant female-advantageous/male-deleterious dominant mutations, given the two thirds of time the X spends in the female germline, thus exposing the advantageous effects more often than the deleterious ones [14]. To account for sex-biased expression, one then evokes modifiers of the sex of expression, reducing expression in the sex in which an allele is deleterious [14]. This we refer to as the sexually antagonistic model. Second, as the X chromosome is inactivated in the male germline, the X has been considered an environment incompatible with the presence of genes needed in spermatogenesis ([15,16], see also [17]). Thus, the traffic of spermatogenesis genes off the X was to be expected, as the ancestral autosome (proto-X) became the X chromosome. The SAXI hypothesis [18] is a fusion hypothesis suggesting that sexual antagonism drove male-biased genes off the X, thereby enabling germline X chromosome inactivation (note that this hypothesis presumes the X chromosome to be incompatible with male-advantage genes although this need not be true).

Both the above models can claim some support (for review see [3,4]). There is, for example, evidence for a movement off the mammalian X chromosome via retroposition of genes whose retroposed copies are highly expressed in the male germline [19]. This is potentially consistent with the germline inactivation/SAXI model but not the version of sexual antagonism that predicts accumulation on the X of genes biased towards male-specific expression. However, unexpectedly [19], there is also evidence for a loss on the X of genes whose retrocopies function after the time of germline X inactivation (i.e., when the X is active again) [19]. Given this potential direct germline effect, tests of the logic of Rice’s hypothesis are best done when considering somatic tissues. While there is evidence that the mouse X is enriched for sex-biased genes not subject to the meiotic sex chromosome inactivation [20], the evidence is somewhat contradictory. For example, one report claims an excess of female-biased gene expression and a dearth of genes biased towards male-specific expression [21]. Another report finds evidence for an enrichment of male-specific genes [22]. In *Drosophila*, genes expressed in male-specific accessory-gland, but not testis, are excluded from the X [23–25].

Here, following a proposal of Vicoso and Charlesworth [26], we wish to suggest that there is a further, potentially complementary, simple yet powerful, driving force for the evolution of gene content on the X chromosome. This force stems from the fact that in males the X is haploid-expressed. This might limit the maximal expression of any X-linked gene. Put simply, when transcription rates are potentially high, if there are two parallel sites for transcription (diploid expression on autosomes), the net rate of production can be higher than if there are transcriptional traffic jams on the haploid-X. The same limitation is not of great importance if the rate of transcription is not limiting. Thus, we expect the X chromosome to be a non-optimal environment for genes with very high maximal levels of expression. Such high levels of expression cannot be readily achieved, owing to transcriptional traffic jams, with all gene expression running through one promoter, as opposed to two promoters in diploid-expressed autosomal genes. X inactivation in female mammals exacerbates the problem. We call this model the “weak X” or traffic jam model.

The precise expectations may depend on the mechanism of dosage compensation. In fruit flies, for example, the X chromosome in males is hyper-transcribed [27,28]. This, it has been argued, will make it hard to increase the expression level even more if there is an upper limit to the rate of transcription, as suggested by Vicoso and Charlesworth [26]. As male-biased expression commonly comes about through increased expression in males, this force alone is enough to explain the absence of male-biased gene expression on the *Drosophila* X [26]. Similarly, Bachtrog et al. [29] find evidence that *Drosophila*’s mode of dosage compensation restricts the ability to further up-regulate X-linked genes. More recent work suggests that genes expressed in male-specific accessory-gland, but not testis, are excluded from the X in *Drosophila* [23–25] because of expression limits on the X.

If, however, the fly X is truly hyper-transcribed, it is an environment for the most part compatible with high maximal expression, just not one readily capable of increasing it still further. Indeed, Vicoso and Charlesworth [26] find no evidence that maximal expression on the X is lower than that on autosomes. The hyper-transcription from the fly X contrasts somewhat with the situation in mammals (as best as it is currently understood). In mammals, it was for a long time believed that the X in males is also hyper-transcribed to compensate for the loss of expression on the decaying Y chromosome [30]. However, two recent analyses indicate that, compared with the expression level of the ancestral genes prior to the formation of the X chromosome (as opposed to the consideration of the current X to autosomal expression ratio), the extant X-linked genes have not increased their expression levels [31,32]. A recent proteomics-based analysis supports this finding [33]. Rather, autosomal genes that partake in protein–protein interactions with X-linked counterparts appear instead to have reduced their expression levels [31]. There is also evidence that some X-linked genes associated with protein complexes have increased their expression [32,34].

Assuming no or limited increase in the expression level of X-linked genes, this suggests a simple explanation for the fortune of genes as the diploid proto-therian X evolved into the haploid-X. Unlike in fly (with hyper-transcription), any gene that had high maximal expression on the proto-mammalian X could not sustain this. If a reduction in dosage was cost-free, then no further adaptation was needed. If a reduction in a gene’s dosage was not cost-free, then an adaptation of some variety might have been required. This might mean divestment of some of a gene’s function to autosomal genes, possibly mediated by changed expression of compensating paralogs or the creation of such paralogs, if not already present. We thus expect a net movement away from (or avoidance of) the X chromosome for genes with high maximal expression. This could, in principle, explain why highly expressed germline genes are moved away from the X, even if there is no germline X inactivation during their time of activity, an observation previously posed as unexpected [19]. If broad expression tends to be coupled with high maximal expression, it might also possibly explain why genes tend to be more tissue-specific on the X chromosome (Table 1) [30,35].

**Table 1:** A lower breadth of expression on the X is observed both in normal and diseased samples in human as well as in mouse.

Sample type	The mean breadth of expression on the X chromosome	The mean breadth of expression on the Y chromosome	The mean breadth of expression on autosomes
Human tissues	0.21 (*p* < 2e-16)*	0.07 (*p* = 3.1e-10)*	0.30
Human primary cells	0.18 (*p* < 2e-16)*	0.05 (*p* = 1.9e-09)*	0.27
Human cancer cell-lines	0.21 (*p* < 2e-16)*	0.05 (*p* = 4.2e-07)*	0.31
Mouse samples	0.23 (*p* < 2e-16)*	0.2 (*p* = 0.085)*	0.33

* *p-*values for Wilcoxon tests in comparisons against the breadth of expression of autosomal genes in the same types of samples are given.

Here, we seek to test these models. To this end, we employ an exceptional expression resource, Functional Annotation of the Mammalian Genome (FANTOM5), and in addition, a merge of FANTOM5 with The Encyclopedia of DNA Elements (ENCODE). FANTOM5 [36] is an extensive atlas of mammalian expression patterns at a single-nucleotide resolution level [37], including libraries from 179 human tissues, 513 isolates of primary cells, and 260 cancer cell-lines, generated using Cap Analysis of Gene Expression (CAGE) technology [38]. Unlike microarrays, CAGE is not limited to preselected features, and it samples the entire genome space in an unbiased fashion. ENCODE [39] is a detailed atlas of regulatory elements. Although ENCODE experiments were performed on separate cell-lines, standardized laboratory protocols and a unified analytical pipeline [40] allow one to merge ENCODE data into a single meta-dataset [41,42].

This work is part of the FANTOM5 (Functional Annotation of the Mammalian Genome 5) Project. Data downloads, genomic tools, and copublished manuscripts are summarized at http://fantom.gsc.riken.jp/5/.

## Results

### The Maximal Expression Level of X-Linked Genes Is Far Below That of Autosomal Genes

**Fig 1:**
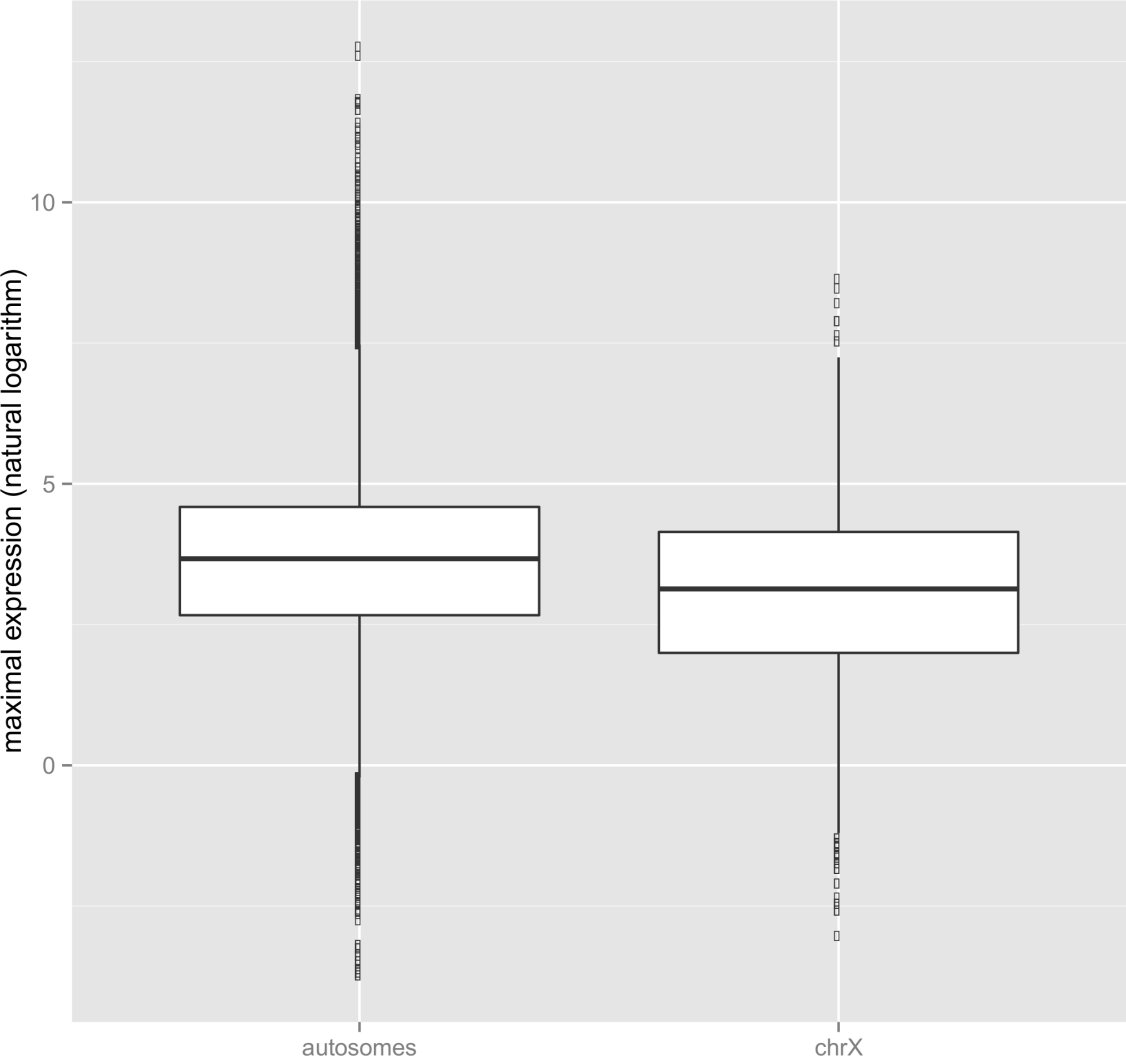
A lower maximal expression level on the X chromosome. This figure shows maximal expression levels for autosomes and the X chromosome. Maximal expression is defined as transcript’s maximal expression level (in TPM) in any of the FANTOM5 human tissues. The underlying data can be found at http://fantom.gsc.riken.jp/5/data/ and in Dryad Digital Repository (doi:10.5061/dryad.p4s57) [43].

A prediction of the “weak X” model is that the upper limit for the highest attainable level of gene expression for X-linked genes should be lower than the maximal attainable for autosomal genes. Consistent with this idea, the average maximal expression on the human X chromosome is three times lower than on autosomes, that is to say 87 versus 261 tags per million (TPM) (Fig 1, Table 2). The difference is highly significant (a Wilcoxon test, *p-*value < 2.2e-16; randomization test’s *p-*value = 0 based on 1 million random subsamples in a test designed to measure the probability of obtaining similarly skewed, i.e., deviant from null, the mean maximal on the X by chance; see Methods). Median maximal expression on the X is also lower than on autosomes: 23 versus 39 TPM. In human tissues, the absolute maximal expression on the X chromosome (highest for any gene in any tissue) is 5604 TPM (brain expressed, X-linked 1 [BEX1], NM_018476, expressed in the medial temporal gyrus library). In contrast, the maximal expression on autosomes is over 62 higher at 3.48e+05 (hemoglobin, beta; NM_000518 expressed in blood; Table 3).

**Table 2:** The X chromosome has a limiting cap on maximal expression.

Basic statistics of maximal expression											
	Maximal expression for all transcripts (in TPM)				Mean maximal expression for housekeeping transcripts, in four definitions (in TPM)						
	Mean	SD	Median	Max	BoE > 0.66		BoE > 0.75	BoE > 0.85		BoE > 0.95	
Autosomes	261	3,672	39	348,120	371		399	483		892	
Chromosome X	87	293	23	5,604	281		289	346		735	
*p-*value (Wilcox)	< 2.2e-16	NA	NA	NA	0.3391		0.0133	0.0045		0.0113	
The quantiles of maximal expression (all transcripts)											
	100.0	99.999	99.998	99.997	99.996	99.995	99.994	99.993	99.992	99.991	99.990
Autosomes	348,120	24,249	13,367	10,416	8,562	7,260	6,392	5,819	5,303	4,883	4,637
Chromosome X	5,604*	3,106	2,645	2,361	2,255	1,956	1,946	1,858	1,834	1,828	1,759
The quantiles of maximal expression (only tissue-specific transcripts–BoE < 0.33)											
	100.0	99.999	99.998	99.997	99.996	99.995	99.994	99.993	99.992	99.991	99.990
Autosomes	295,980	31,831	14,264	9,992	7,419	6,034	5,016	4,334	3,798	3,404	3,095
Chromosome X	1,402	1,301	1,101	869	828	794	773	594	570	525	492
The distribution of top maximally expressed genes (all genes)											
	0.1%	0.2%	0.3%	0.4%	0.5%	0.6%	0.7%	0.8%	0.9%	1%	10%
Autosomes	29	57	83	111	141	164	191	218	244	270	2,171
Chromosome X	0	0	0	0	0	2	2	3	3	3	65
*p-*value (Fisher’s test**)	0.6306	0.1746	0.08254	0.02438	0.007528	0.0707	0.02617	0.0377	0.0206	0.0077	0.007957
The distribution of top maximally expressed genes (only tissue-specific genes–BoE < 0.33)											
	0.1%	0.2%	0.3%	0.4%	0.5%	0.6%	0.7%	0.8%	0.9%	1%	10%
Autosomes	23	41	54	69	83	94	104	114	122	134	736
Chromosome X	0	0	0	0	0	0	0	0	0	0	16
*p-*value (Fisher’s test**)	0.6254	0.2644	0.1136	0.07939	0.03404	0.0237	0.009947	0.0067	0.0045	0.0031	0.000311

NOTE: The results shown correspond to FANTOM5 human tissue expression data. Maximal expression is the greatest numerical value attained (in tags per million) for each transcript in any single library of the collection. Thus, the maximum does not arithmetically depend on the breadth of expression (BoE) or average expression (although it correlates with them).

* Mean maximal expression on the X is 62-times lower than on autosomes.

BoE, breadth of expression; SD, standard deviation; TPM, tags per million

** Fisher’s exact test’s *p-*values were calculated in a two-by-two contingency table where the observed distribution of genes with the expression level in the top quantile were compared against the random expectation set by the ratio of the total set of 17,989 autosomal genes and 759 X-linked genes (those numbers were 10,929 and 550, respectively, for tissue-specific genes).

**Table 3:** The top 25 autosomal genes by maximal expression, with corresponding tissues of expression.

Transcript name	RefSeq ID	The expression level detected (in TPM)	The corresponding tissue of expression
Transthyretin (TTR)	NM_000371	44,862	Medulla oblongata, adult
Proline-rich protein BstNI subfamily 3	NM_006249	47,975.3	Parotid gland, adult
Actin, alpha 1	NM_001100	58,070.1	Skeletal muscle, adult
Trypsin 1	NM_002769	61,176.9	Pancreas, adult
Actin, alpha 1	NM_001100	63,442.3	Artery, adult
Statherin	NM_003154	67,021.2	Parotid gland, adult
Carboxypeptidase A1	NM_001868	68,343.6	Pancreas, adult
Carboxypeptidase B1	NM_001871	70,360.8	Pancreas, adult
Semenogelin 1	NM_003007	70,653.1	Seminal vesicle, adult
Prolactin	NM_000948	72,523.8	Pituitary gland, adult
Albumin	NM_000477	78,347.1	Liver, adult
Statherin (STATH), transcript variant 2	NM_001009181	80,362.5	Submaxillary gland, adult
Statherin (STATH), transcript variant 1	NM_003154	80,362.5	Submaxillary gland, adult
Prolactin (PRL)	NM_000948	82,030.2	Pituitary gland, adult
Chymotrypsinogen B2	NM_001025200	89,742.1	Pancreas, adult
Serpin peptidase inhibitor	NM_000295	95,007.2	Liver, adult
Colipase, pancreatic	NM_001832	112,774	Pancreas, adult
Semenogelin I	NM_003007	112,860	Ductus deferens, adult
Histatin 3	NM_000200	126,071	Parotid gland, adult
Serpin peptidase inhibitor	NM_000295	127,057	Liver, fetal
Statherin, transcript variant 2	NM_001009181	130,889	Salivary gland, adult
Statherin, transcript variant 1	NM_003154	130,889	Salivary gland, adult
Proline-rich protein BstNI subfamily 4	NM_002723	136,988	Parotid gland, adult
Submaxillary gland androgen regulated protein 3B	NM_006685	295,980	Salivary gland, adult
Hemoglobin, beta	NM_000518	348,120	Blood, adult

Note that maximal expression is the greatest numerical value attained (in TPM) for a transcript across all relevant CAGE libraries and thus, unlike the mean (or the median) expression, maximal expression does not greatly suffer from method problems relating to cutoffs to call a gene expressed or not. As then expected, the effect is robust to the exclusion of “non-expressed” genes, with the average maximal expression on the X and autosomes at 122 and 321 TPM respectively (a Wilcoxon test, *p-*value = 2.96e-12) for genes with a minimum maximum expression level of 10 TPM. The result is also robust when considering maximal expression per gene (where multiple transcripts from one gene are amalgamated by averaging or summing their expression levels), instead of maximal expression per transcript (S1 Table).

An analysis of the behavior of the large pseudoautosomal region (PAR1), with its 24 genes, is broadly compatible with the view that the maximal expression constraint is specific to the haploid part of the X chromosome (S2 Table). Definitive statements should, however, not be made, owing to the limited sample size. Indeed, if we randomly select 24 autosomal genes and ask how often these are significantly different in their mean maximal expression to genes on the haploid part of the X, then in less than 50% of randomizations do we detect any effect (S2 Table), while the comparison of all autosomal genes to all X-linked genes is unambiguous. This caveat aside, we note two things. First, the mean maximal expression for PAR1 genes is higher than that of haploid-X genes, this being on the edge of significance (94 versus 86 TPM, *p* = 0.059, a Wilcoxon test). However, additionally, we note one peculiarity, this being that the average breadth of expression (BoE) of PAR1 genes is rather low (S2 Table). As the low breadth of expression is likely to correlate with low maximal expression (for sampling reasons alone, see below), we also ask about the maximal expression of PAR1 genes controlling for the breadth of expression. This we do by performing a *loess* regression of maximal expression predicted by the breadth of expression for all genes on the X chromosome (PAR1 included) and then calculating the residuals. A positive residual implies a maximal level of expression that is high given the underlying breadth of expression. We find that PAR1 genes have on average positive residuals (the mean of 54) whilst the haploid-X-linked genes have weakly negative residuals (the mean of -3.27). The two sets of residuals are significantly different (*p* = 0.007 in a Wilcoxon test). Thus, controlling for the breadth of expression, PAR1 genes have higher maximal expression than genes on the haploid-X. Why PAR1 genes have a reduced breadth of expression is unclear, but with just 24 data points, and a tendency for tissue-specific genes to cluster [35], this may be little more than sampling artifact.

### Highly Expressed X-Linked Genes Cannot Easily Increase Their Expression

If high expression of X-linked genes is difficult, then we might expect that lowly expressed X-linked genes might be able to increase their expression more readily than highly expressed ones, the latter having a problem with transcriptional traffic jams. To address this we consider Brawand’s RNAseq dataset [44], presenting expression of orthologous genes across five somatic tissues in males in several primates. Because it includes primate data, the Brawand et al. dataset is better suited to address this aspect of our analysis than FANTOM5. Note that we do not wish to determine whether immediately after the formation of the X, the X was up-regulated; rather we wish to know whether during a more normal phase of expression evolution, genes on the X are constrained in their ability to increase expression.

**Fig 2:**
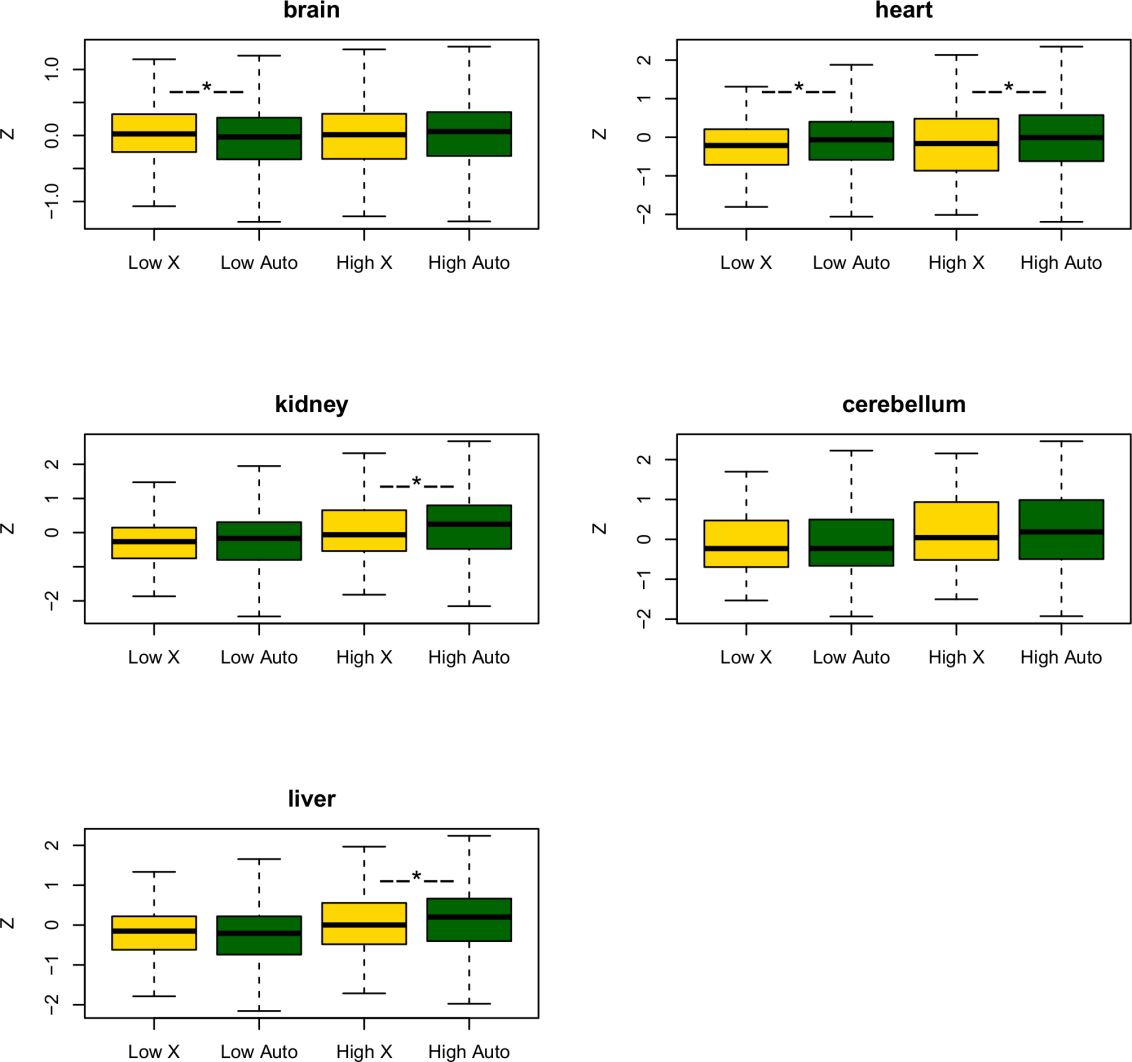
The comparison of change in gene expression (Z) since the human-Chimpanzee common ancestor for five somatic tissues. Genes are divided into X-linked (yellow) and autosomal (green). In turn, they are split into a half with low expression in the ancestor (*low*) and a half with high expression (*high*). Genes with no expression in the ancestor are excluded from this analysis (but this makes no qualitative difference). In all instances, the high-expression X-linked genes have a lower median Z-score than high-expression autosomal genes, this being significant in three instances using a Mann Whitney U test (shown as *). The combined *p-*value is highly significant (see main text). There is no consistent trend for the low-expression genes. The underlying data can be found at http://fantom.gsc.riken.jp/5/data/ and in Dryad Digital Repository (doi:10.5061/dryad.p4s57).

Here, we used a Bayesian approach to infer the ancestral expression state in the human/Chimpanzee common ancestor. We then used the ancestral state to define the change in expression from the ancestor to the current human expression level, expressing this as a Z-score. The Z-score factors in noise in both the estimation of current levels and the ancestral state. Positive Z-scores imply increases in expression since the common ancestor. We excluded from the analysis genes with no expression in the ancestor in any given tissue, as these are most likely unexpressed genes (although this exclusion makes no qualitative difference). We calculated a tissue-specific *p-*value comparing the Z-scores for X-linked genes and Z-scores for autosomal genes via a Wilcoxon test for each tissue (Fig 2). We then combined these scores using Fisher’s method to generate a single *p-*value for each test. Note that our metric of expression change is not in terms of fold-change as this would almost certainly bias towards finding a larger effect for lowly expressed genes (it is easier to double expression of a lowly expressed gene than it is of a highly expressed gene). Instead, we take a more conservative measure, asking about absolute change in standard deviation units, not least because the thesis we are testing concerns the difficulties in increasing the absolute expression level.

The median expression level for X-linked genes in the Brawand et al. dataset is much lower than that for genes on autosomes (the mean ratio of median-X to median-autosome equals 0.58). This confirms a lower expression on the X previously claimed for this data [31]). More importantly, Z-score is lower for the X in each of the five tissues, the net difference being significant (chi-squared = 30.9, *d*.*f*. = 10, *p* < 0.001). We then split the autosomal and X-linked genes into two groups: a highly expressed half (according to the expression level in the ancestor, for each chromosome class) and a lowly expressed half (Fig 2). For each of the five somatic tissues, the highly expressed autosomal genes have a greater median Z-score than the highly expressed X-linked genes (Fisher’s method for combination of *p-*values, chi-squared = 26.9, *d*.*f*. = 10, *p* < 0.005). By contrast, for the lowly expressed half of the genes, the X has a higher median Z-score than autosomes have in two cases and a lower median Z-score in three. In two tissues, the effect is significant via a Wilcoxon test, one where the X has a higher Z-score (brain) and one where autosomes have the higher Z-score (heart). In sum, the data support the notion that highly expressed X-linked genes typically do not increase their expression as much as highly expressed autosomal genes, but the same is not true for lowly expressed genes. These results are as predicted by the traffic jam hypothesis.

### Intolerance of High Expression Explains, in Part, the Tissues within which X-Linked Genes Are Rarely Expressed

**Fig 3:**
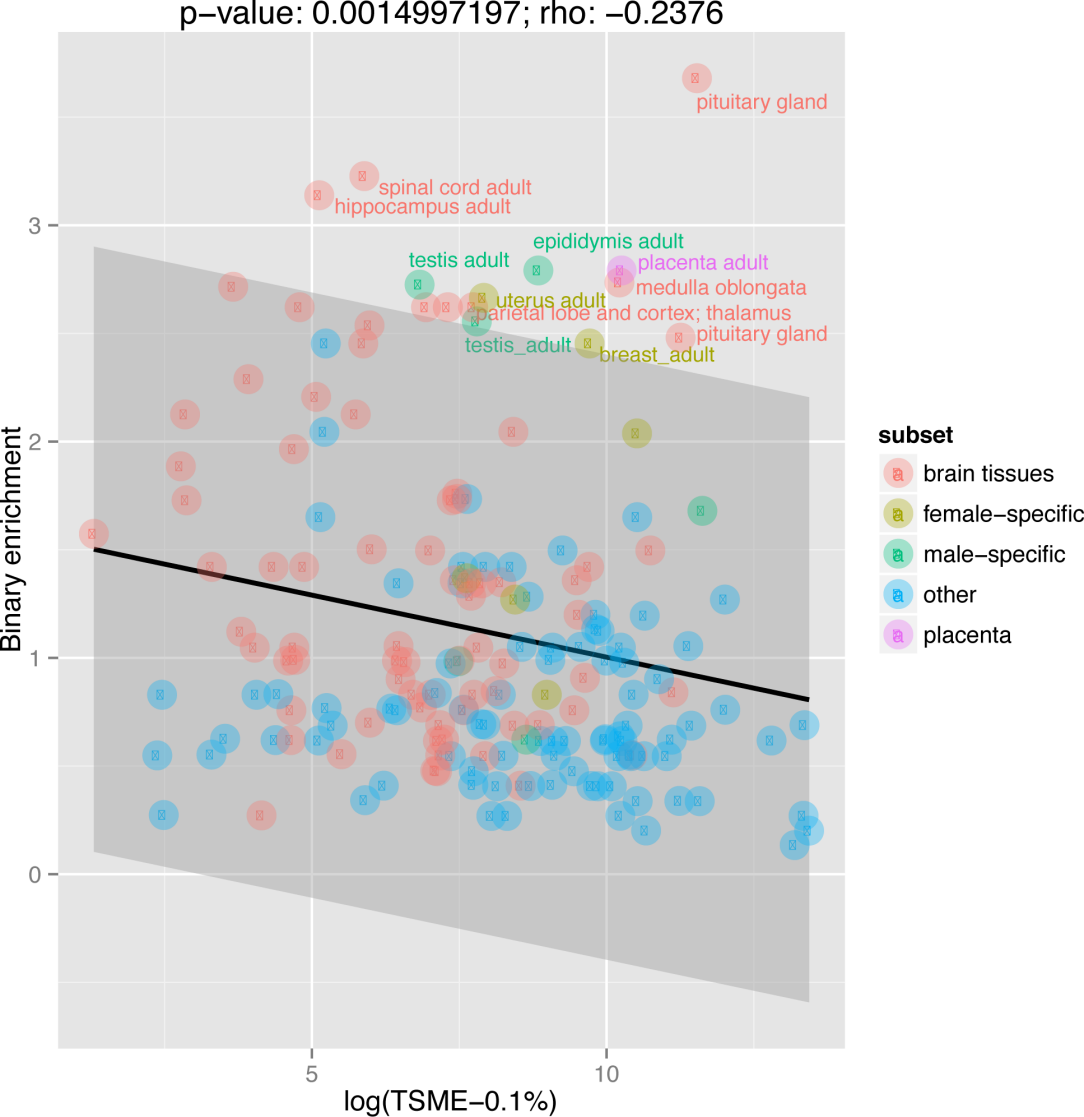
A correlation between tissue-specific maximal expression (TSME) and binary enrichment on the X chromosome. This figure shows a scatterplot where each data point is a FANTOM5 library (points are colored-coded to highlight brain tissues, sex-specific tissues, and the placenta). *X*-axis corresponds to the average maximal expression of given tissue’s top 0.1% preferentially expressed genes (i.e., TSME, using a logarithmic scale). *Y*-axis corresponds to binary enrichment. The strength of the Spearman correlation and *p-*values are annotated with text above the figure panel. Data points that have standardized residuals more than 1.96 standard deviations (highlighted as grey area) from the linear regression line (which is plotted in black) have their names annotated with text. The underlying data can be found at http://fantom.gsc.riken.jp/5/data/ and in Dryad Digital Repository (doi:10.5061/dryad.p4s57).

If intolerance of genes with high maximal expression shapes the X chromosome, it should be also the case that tissues with highly expressed tissue-specific transcripts should be avoided on the X. To test this, we calculated the average expression for a selected set of the top 1% or 0.1% of most tissue-specific genes for each CAGE library (these metrics are called tissue-specific maximal expression or TSME-1% and TSME-0.1%). These metrics we assume to reflect the maximal expression level needed in any given tissue to carry out its tissue-specific physiological functions. We chose two cutoffs to make the analysis more robust. Our expectation is that tissues requiring high expression of their tissue-specific genes (that is high TSME), such as glands or specialized epithelia, should be also those whose specific genes are underrepresented on the X chromosome. To estimate over- and under-representation on the X, we define the metric of binary enrichment for a set of genes as the fold enrichment on the X against the random expectation based on the X-to-autosomal ratio of the total human gene set (see the Methods section *Defining enrichment metrics*, for the detailed definition of binary enrichment). A tissue with high binary enrichment would have most of its tissue-specific genes on the X chromosome. Next, we correlate the TSME measures with the metrics of tissue’s binary enrichment on the X chromosome for the matching sets of top 1% or 0.1% tissue-specific genes (Fig 3). Both TSME and binary enrichment are compatible metrics focusing on a tissue’s uniquely expressed and preferentially expressed genes (which are the ones we are interested in in this test).

If tissues with high-level maximal expression of tissue-specific genes are tissues for which tissue-specific expression is avoided on the X chromosome, then we expect a negative correlation between X-enrichment and TSME. As expected, we indeed see such a correlation (although it is only statistically significant for the top 0.1% of each tissue’s preferentially expressed genes with *rho* = -0.1328 and *p* = 0.0788 for TSME-1%; and *rho* = -0.2376 and *p* = 0.001499 for TSME-0.1%). The above correlations (i.e., between TSME and binary enrichment metrics) are stronger when sex-specific tissues (both male and female) are removed from all stages of calculations (*rho* = -0.1874 and *p* = 0.01628 for TSME-1%; *rho* = -0.2843 and *p* = 0.0002253 for TSME-0.1%). This suggests that this result is not explained by sexual antagonism or the germline inactivation hypothesis.

**Fig 4:**
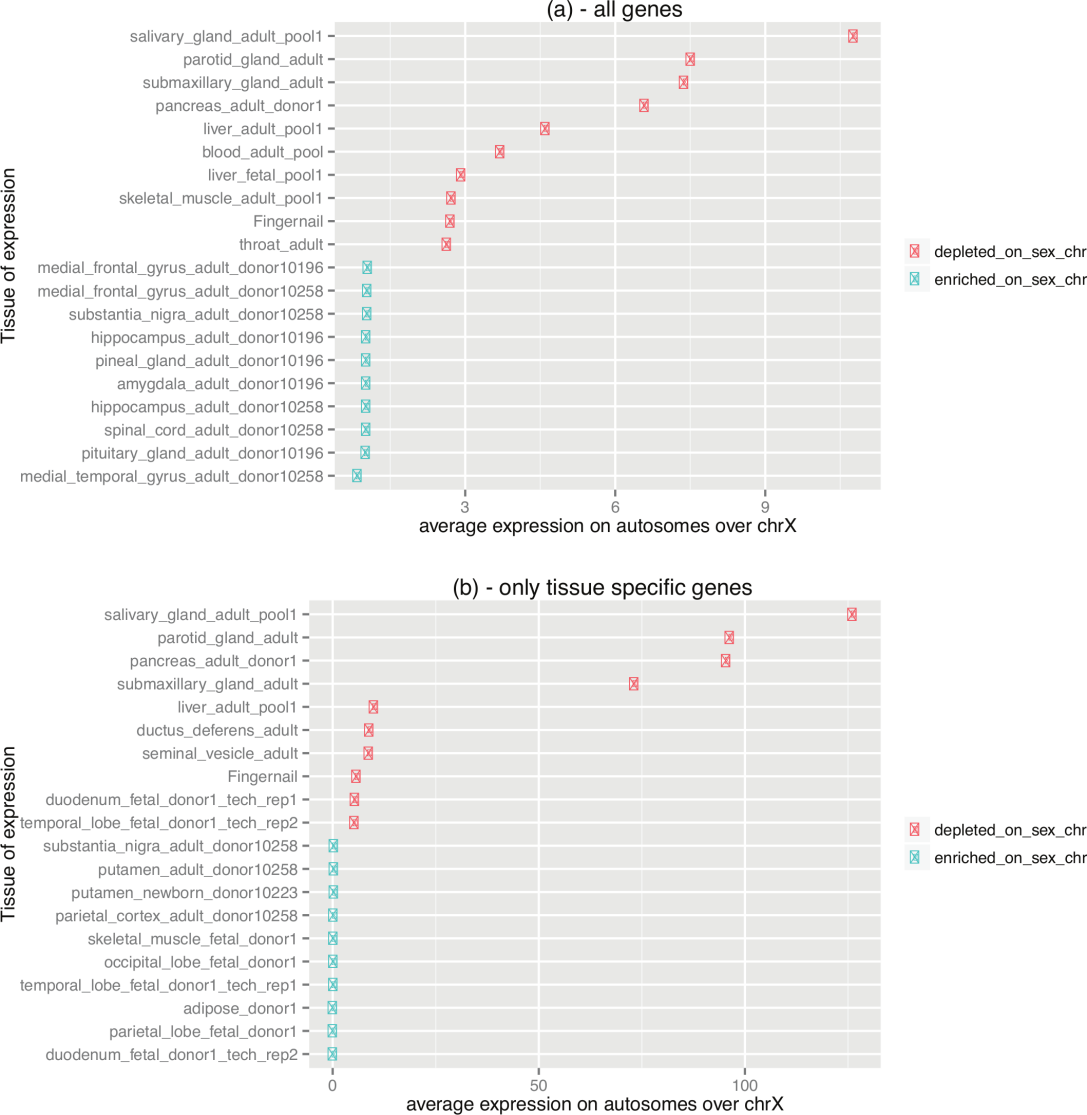
Analog enrichment in expression on the X chromosome. This figure consists of two panels identifiable as *a* and *b*. Each panel shows the ratio of average (*per locus*) expression on autosomes over that observed on the X chromosome (if the ratio was higher than one given tissue was enriched in expression on autosomes). Panel *a* shows data for all genes, panel *b* shows data only for tissue-specific genes (i.e., these with the breadth of expression lower than 0.33). Only the top ten over-represented and the top ten under-represented tissues are shown. Brain subsets are clearly most X-enriched tissues. Exocrine gastrointestinal glands, in contrast, are the most X-depleted tissues. The underlying data can be found at http://fantom.gsc.riken.jp/5/data/ and in Dryad Digital Repository (doi:10.5061/dryad.p4s57).

In asking about the enrichment of tissue-specific genes on the X chromosome after controlling for a tissue’s expression level, our model provides an explanation for the patterns of tissue enrichment not obviously accounted for by other models. That is to say, tissues whose specific genes are under-represented on the X tend to be secretory or rich in structural proteins. These are, for example, the exocrine glands of the gastrointestinal tract (i.e., the submaxillary gland, the parotid gland, the pancreas, and the liver) or highly keratinized tissues such as the tongue, throat, or esophagus (analog enrichment in Fig 4, S3 Table; binary enrichment in S4–S6 Tables). These are also likely to be highly transcriptionally active tissues.

An analysis of the most highly expressed genes on autosomes and the X reinforces the same conclusion regarding the avoidance of tissue associated with active secretory processes on the X. There are 401 autosomal transcripts/expression sites with higher maximal expression than the maximum of all values on the X chromosome, which is 5,604 TPM (these transcripts and expression sites are listed in S7 Table). These 401 transcript-in-tissue data points are derived from 159 distinct transcripts. It should be noted that not only this tail of transcripts with high-maximal expression is absent from the X, but also X’s mean maximal expression is 3-times lower—see the first Results section). The observation of the biased autosomal/X distribution of these highly maximally expressed transcripts is statistically significant (Fisher's exact test for count data: *p* = 0.00143). The 159 transcripts were also strongly biased functionally, with many secreted proteins, protease and peptidase inhibitors, muscle proteins, liver enzymes, coagulation factors, lipid transporters, digestion enzymes, hormones, and proteins involved in reproduction (for details and *p-*values see S8 Table). In contrast, 159 top maximally expressed X-linked transcripts appear to not be associated with secretory processes (S9 Table). Instead they are linked to functional terms for melanoma antigen E (MAGE) tumor-specific antigens, actin binding, association with the mitochondrial membrane, endoplasmic reticulum and microsome, erythrocyte differentiation, hemopoiesis, nucleosome assembly, DNA packaging, neuron development, neurogenesis, ribosome and cell death (for details and *p-*values see S9 Table).

It is interesting to ask a complementary question: one about libraries under-represented in the expression domain of X-linked genes in comparison to autosomal genes. To this end, we define the metric of binary exclusion, which asks about the autosomal-to-X ratio for all genes expressed (i.e., "on") in a given tissue (for details see the Methods section *Defining enrichment metrics*). Binary exclusion is complementary, but not exactly the opposite of binary enrichment, as the former focuses on all genes expressed in a given tissue, while the latter asks only about tissue-specific genes. When we analyze histograms of binary exclusion (S1*A* Fig, see also S10–S12 Tables), only 7 tissues are more than 1.96 standard deviations over the mean degree of exclusion (the mean = 1.48, SD = 0.24). These tissues are the fingernail, cruciate ligaments, the adult pancreas, skin of the palm, the Achilles tendon, the inferior rectus of the eye, and tongue epidermis—suggesting preferential exclusion only for secretory tissues, or tissues extremely rich in highly expressed structural proteins. Moreover, it is striking that all tissues except the substantia nigra, a tiny brain structure located in the midbrain, are excluded to some extent. This suggests that the lowering of the breadth of expression on the X is a universal phenomenon affecting all tissues, rather than having its origin in exclusion from any particular type of tissues such as sex-specific or mammalian-specific tissues.

### The Impoverishment of Housekeeping Genes on the X Is Explained by the Avoidance of High Maximal Expression

#### X-linked genes tend to be more tissue-specific

Prior evidence suggests that X-linked genes are relatively tissue-specific [30,45]. The FANTOM5 data strongly support this conclusion (Table 1, Table 4 and S1 Table). The breadth of expression was defined as the fraction of samples in which a given gene was “on” (that is expressed at more than 10 TPM). The motivation for the choice of the cut-off was described previously [46]. Confirming and extending prior claims [45], a lower breadth of expression on the X is observed in all sample categories in human, mouse, and rat (Table 1). The average breadth of expression on autosomes (*n* = 29,400) is 0.3, versus 0.21 (*n* = 1,433) on the X (Wilcoxon rank sum test *p* < 2.2e-16). The fraction of housekeeping transcripts (the breadth of expression >66%) is 13.6% on the X versus 21.5% on autosomes (Table 4). To control for the distant possibility that the lower breadth of expression on the X was due to a higher fraction of non-expressed artifactual RefSeq transcripts (that is those with expression signal lower than 10 TPM), we verified that the same result is found both when all genes are considered and when only genes with detectable expression are taken into account (S2 Fig).

**Table 4:** A lower breadth of expression (BoE) on the X is mostly due to the exclusion of housekeeping transcripts.

The number of transcripts			Chromosomal location:autosomes (A), the X chromosome (X).
Housekeeping	Intermediate	Tissue-specific	
195 (14%)	155 (11%)	1,083 (76%)	X (all genes)
195 (19%)	155 (15%)	658 (65%)	X (only expressed genes)
6,328 (22%)	4,381 (15%)	18,691 (64%)	A (all genes)
6,328 (27%)	4,381 (18%)	13,170 (55%)	A (only expressed genes)
**+36%**	**+27%**	**-19%**	**percentage shift: A minus X (all genes)**
**+30%**	**+17%**	**-18%**	**percentage shift: A minus X (only expressed genes)**

NOTE: Percentage values in brackets refer to the fractions of row totals and sum up to 100. Percentage values in bold, which do not refer to row totals and do not sum up to 100, refer to the magnitude of the shift in a given gene category (either housekeeping, intermediate, or tissue-specific) between autosomes and the X. For example, +36% was calculated as (22–14)/22 * 100% and signifies that the proportion of housekeeping genes on autosomes was 36% higher than on the X chromosome. The greatest difference between the X chromosome and autosomes is in the category of housekeeping genes (+36% and +30% for all genes and only expressed genes respectively). “Only expressed” are those genes with evidence of expression in the FANTOM5 human tissue set (TPM > 10).

#### On the X chromosome, a low breadth of expression corresponds to a low number of transcription factor binding sites (TfbsNo) per promoter

**Fig 5:**
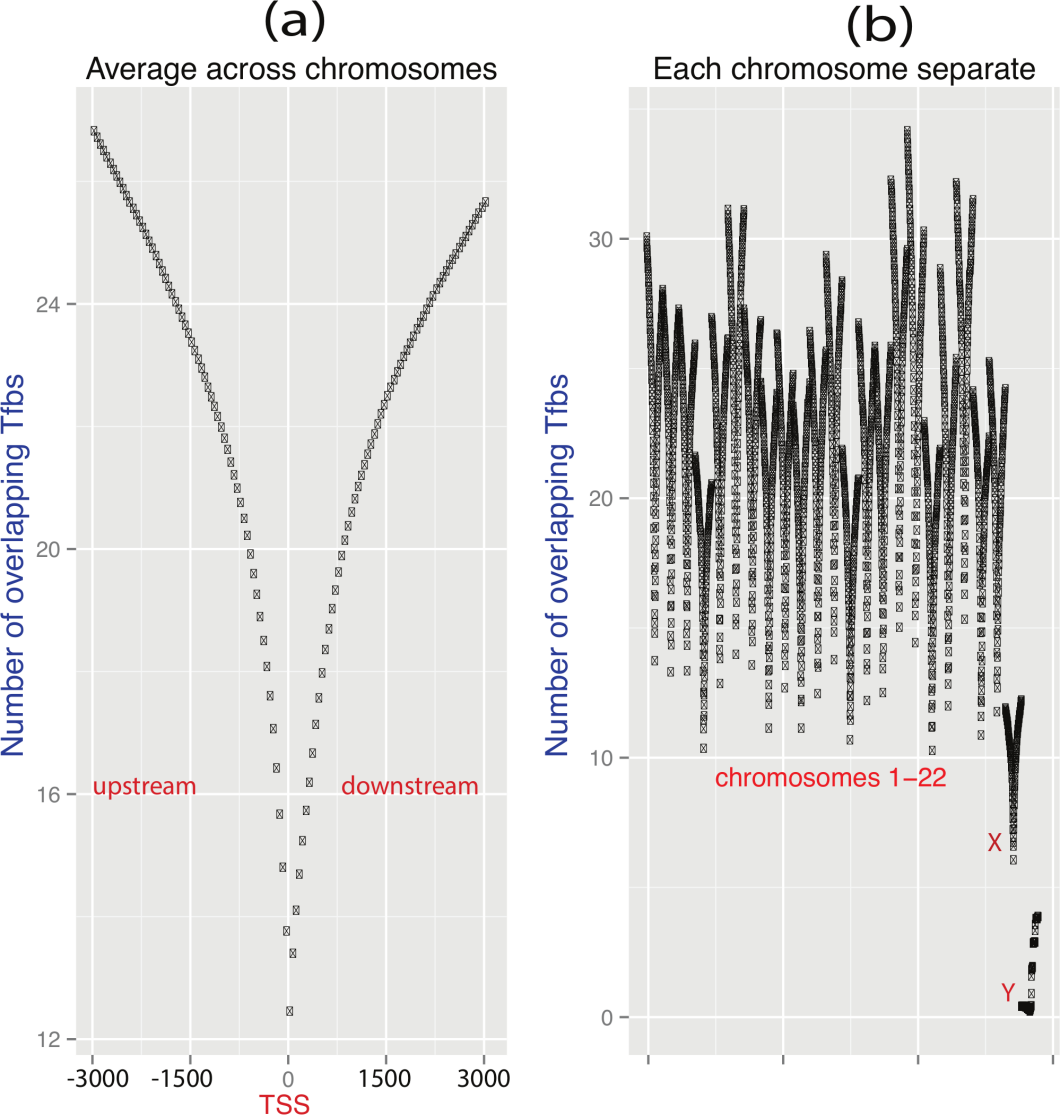
The number of transcription factor binding sites per proximal promoter is higher on autosomes than on sex chromosomes. This figure consists of two parts identified as *a* and *b*. In part *a*, the average number of transcription factor binding sites per promoter in symmetrical windows around transcriptional start sites (TSSes) is shown. The plots have a characteristic shape of the V-sign. On the *x*-axis of panel *a*, values from negative 3 kbps to zero signify positions upstream TSSes (negative values signify positions downstream the TSS). In part *b*, V-sign-shaped curves are plotted separately for each chromosome (and the *x*-axis corresponds to the order of chromosomes from 1 to 22 plus the X and Y). The curves are similar between autosomes, but TfbsNo is lower for sex chromosomes. The underlying data can be found at http://fantom.gsc.riken.jp/5/data/ and in Dryad Digital Repository (doi:10.5061/dryad.p4s57).

The X chromosome thus appears enriched for genes of narrow (S2 Fig) and low maximal expression (Fig 1). How might this be controlled? Previously, we have shown that the breadth of expression is strongly predictable from the knowledge of TfbsNo [46]. Might then a loss of transcription factor binding sites explain in part the reduced breadth of expression of X-linked genes (assuming that ancestrally X-linked genes had an average autosomal TfbsNo)? Here, we then ask whether the TfbsNo on the extant X is lower than on autosomes. To quantify TfbsNo on autosomes and the X, we explored a range of window sizes for detecting ENCODE transcription factor binding sites in promoters: from a hundred base pairs to ten thousand base pairs (see Fig 5*A*, S13 Table). The numbers of mapping transcription factor binding sites increased continuously with the size of the promoter window, but was always lower on the X than on autosomes (S13 Table). The plot of the density function formed a characteristic V-shaped curve (Fig 5*A*). For all window sizes, TfbsNo on the X and Y was lower in comparison with that on autosomes (S13 Table, Fig 5*B*). We also observed a lower overall density of transcription factor binding sites on sex chromosomes per kilobase (kb) of DNA (S3*A–*S3*D* Fig) than would be expected by the general correlation between TfbsNo and gene number (S4*F* and S4*H* Fig). To our knowledge, this is the first report of a lower TfbsNo on sex chromosomes.

#### The reduced breadth of expression on the X is not owing to its unusual gene content

**Fig 6:**
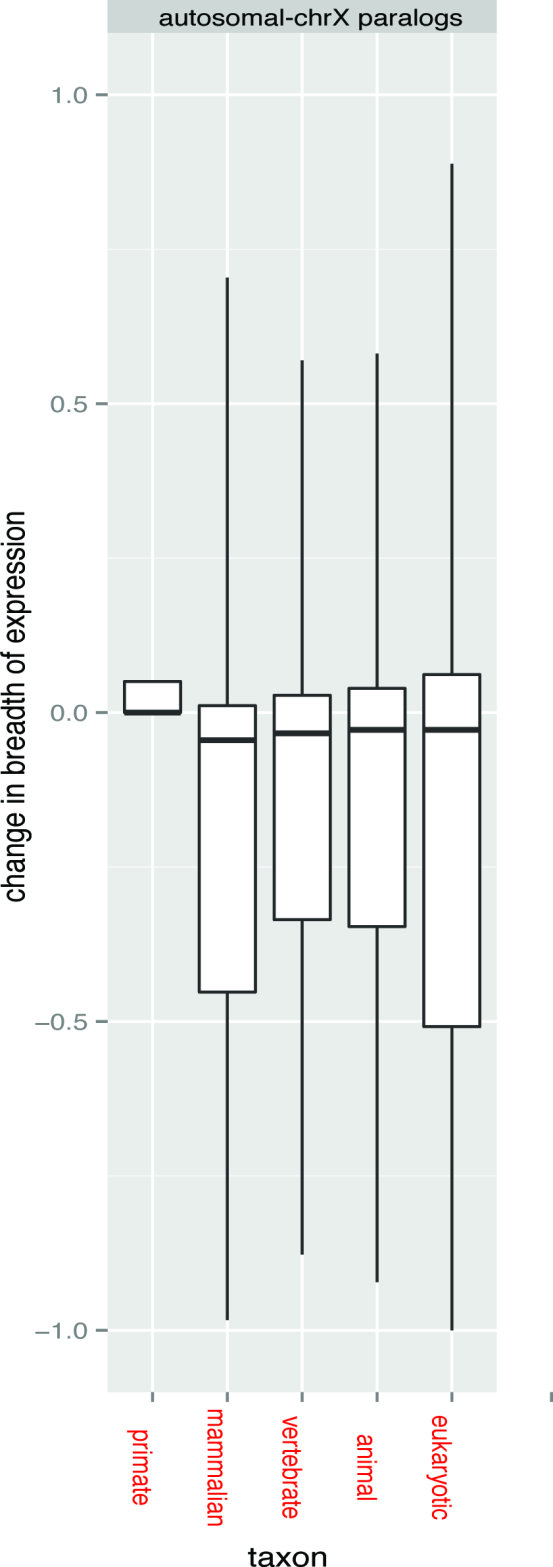
A shift in the breadth of expression for split pairs of autosomal-X paralogs. This figure shows barplots for the shift in the breadth of expression (ΔBoE) depending on the taxon of duplication for autosomal-X paralogs. The critical result is that all groups except primates were shifted significantly below zero (Wilcoxon one-sided test *p-*values are given brackets): primate (*p* = 0.639), mammalian (*p* = 2.479e-12), vertebrate (*p* = 9.178e-13), animal (*p* = 1.046e-07), eukaryotic (*p* = 0.00087). The differences between groups were not statistically significant after multiple-testing correction, but it was not the point of this analysis to show any differences between the taxa. For figure clarity, we do not show these data, but as expected (as these are non-directional comparisons), the average ΔBoE is close to zero for autosomal-only (same or different chromosome), X-only, and Y-only duplications, regardless of age. The underlying data can be found at http://fantom.gsc.riken.jp/5/data/ and in Dryad Digital Repository (doi:10.5061/dryad.p4s57).

Might the lower breadth of expression reflect something peculiar about the functional classes of genes on the X? To address this, we ask about the breadth of expression, maximal expression, and TfbsNo of X-linked genes with autosomal paralogs, thereby controlling for gene class. Importantly, for gene families with X and autosomal representatives, X-linked copies have a lower breadth of expression and a lower maximal expression compared to their autosomal paralogs (Table 5, Table 6 and Fig 6). This supports the hypothesis that reduced breadth of expression of X-linked genes is not a peculiarity of the genes that happen to be on the X, but rather is a peculiarity of the X itself. As expected, this difference in the breadth of expression between X-linked and autosomal paralogs is also reflected in different numbers of transcription factor binding sites (Table 5 and Table 6). As then also predicted, the difference in the breadth of expression is explained in part by the difference in TfbsNo, this correlation being observed for all types of split autosomal-X paralog pairs (Table 5).

**Table 5:** Proximal promoter changes in the breadth of expression (ΔBoE), the number of transcription factor binding sites (ΔTfbsNo) and the maximal expression (ΔMAXIMAL) have, on average, negative cumulative values for split pairs of X-autosomal paralogs (with the autosomal member of the pair having, on average, a higher breadth of expression, TfbsNo, and maximal expression). PCC is the Pearson correlation coefficient; *rho* is the Spearman rank correlation coefficient.

Retroposition status	ΔBoE	ΔTfbsNo	ΔMAXIMAL MAXIMAL_X_ MAXIMAL_autosomal_	Coexpression	N	The correlation between ΔBoE and ΔTfbsNo	DAVID enriched terms
Both paralogs are retrogenes	-0.16±0.42	-2.52±3.25	-70±134 113±118 184±187	PCC = 0.38±0.36 *rho* = 0.32±0.28	15	PCC = 0.75 *p* = 0.001211	MAGE protein (Interpro, *n* = 4, *p* = 1.4E-6), forebrain development (go-bp, *n* = 2, *p* = 4.4E-2)
Not a retroposition	-0.15±0.38	-3.91±7.94	-79±677 92±312 171±632	PCC = 0.19±0.25 *rho* = 0.25±0.27	850	PCC = 0.27 *p* = 8.882e-16	Mental retardation (sp-pir, *n* = 34, *p* = 1.8E-37), part of plasma membrane (go-cc, *n* = 74 *p* = 1.8E-4), Alport syndrome (sp-pir, *n* = 4, *p* = 5.1E-4), epilepsy (sp-pir, *n* = 7, *p* = 8.3E-4)
Retroposition *auto→X*	-0.47±0.44	-10.42±8.94	-675±2424 32±61 709±2420	PCC = 0.09±0.19 *rho* = 0.10±0.16	40	PCC = 0.63 *p* = 1.101e-05	Transcription (go-bp, *n* = 8, *p* = 6.7E-2), nucleus location (sp-pir, *p* = 5.1E-2, *n* = 14)
Retroposition *X→auto*	-0.34±0.57	-1.57±7.29	-88±308 41±84 129±293	PCC = 0.15±0.28 *rho* = 0.12±0.28	130	PCC = 0.69 *p* < 2.2e-16	MAGE protein (Interpro, *n* = 20, *p* = 7.1E-38)

NOTE: N denotes the number of relevant X-linked transcripts.

DAVID version 6.7 is available at http://david.abcc.ncifcrf.gov.

Autosome-to-X retrogenes are associated with lower maximal expression of retrogenes (the mean of 32±61), than seen for X-to-autosome retrogenes (the mean of 129±293), *p-*value = 6.347e-12 in a Wilcoxon test. Only the youngest paralog pairs are considered.

ΔBoE = BoE_X_—BoE_autosomal_; ΔTfbsNo = TfbsNo _X_—TfbsNo _autosomal_; ΔMAXIMAL = MAXIMAL_X_—MAXIMAL_autosomal_.

**Table 6:** The asymmetric divergence of split autosomal-X paralog pairs: the loss of transcription factor binding sites, a shift towards capped expression (i.e., limited in the maximal level) and a shift towards more tissue-specific expression on the X.

The timing of the duplication event (estimated by phylogenetic timing).	The number of duplication events (i.e., unique nodes in TreeFam phylogenetic trees which are classified as duplications rather than speciation events).					*ΔBoE*	*ΔTfbsNo*	*ΔMAXIMAL*	Expression in selected tissues		
RR	Not-R	*Auto→X*	*X→auto*	Total	*B*	*M*	*F*				
Human	0	0	0	0	0	NA	NA	NA			
Human/Chimpanzee/Gorilla	0	2	1	2	5	0.44	4	45±104	1	1	1
Catarrhini	0	3	4	0	7	-0.43	-6.28	-93±113	7	1	1
Eutheria	6	80	16	57	159	-0.27	-5.38	-49±121	4	1	1
Theria	0	49	2	6	57	-0.28	-3.31	-276±1148	3	1	0
Amniota	1	6	1	0	8	-0.09	-5.70	-245±653	13	1	1
Tetrapoda	0	18	1	7	26	-0.12	-0.41	-87±172	3	2	1
Vertebrata (2R-WGD)	4	509	14	45	572	-0.21	-3.5	-125±1000	2	1	1
Chordata	1	95	2	2	100	-0.12	-4.56	-80±320	2	1	1
Deuterostomia	0	10	0	1	11	0.06	-0.86	14±84	1	1	1
Bilateria	0	125	4	4	133	-0.10	-2.67	-8±387	2	1	1

NOTE: 2R-WGD, 2 rounds of whole genome duplication; RR, both paralogs are retrogenes; not-R, not a retroposition; *auto→X*, autosomal-to-X retroposition; *X→auto*, X-to-autosomal retroposition. *B*, *M*, *F*, stand for enrichment in brain, male, and female-specific expression (the average expression in selected tissues divided by the average expression in all tissues, both in TPM, for all transcripts mapping to genes assigned to the specific taxon of duplication by phylogenetic timing; the *B* set is as defined in S19 Table; *M* and *F* tissue subsets are as defined in Table 7). ± indicates standard deviation.

ΔBoE = BoE_X_ - BoE_autosomal_; ΔTfbsNo = TfbsNo _X_ - TfbsNo _autosomal_; ΔMAXIMAL = MAXIMAL_X_ - MAXIMAL_autosomal_.

Is this correlation between the breadth of expression and TfbsNo a consequence of the preservation of preexisting binding sites formed through block duplications, or might it rather reflect selective remodeling as our model predicts? As retroposed genes do not take their promoters with them, we can address this issue by splitting the paralogs into retroposed and non-retroposed sets. That the correlation is seen for both (Table 5) suggests that the trend is not a passive preservation of preexisting promoters, but rather reflects selected promoter remodelling. Perhaps surprisingly the correlation is if anything stronger for retroposed genes. As ΔMAXIMAL (change in maximal expression between paralogs) correlates much stronger with ΔBoE (*rho* = 0.67, *p* < 2.2e-16) than with ΔTfbsNo (*rho* = 0.22, *p* = 7.752e-15), we suggest that for non-retroposed pairs the limiting cap on maximal expression (rather than ΔTfbsNo) is the more direct force limiting the breadth of expression.

#### Sexual antagonism, reduced recombination, retroposition, or germline X inactivation do not explain the reduced breadth of expression on the X

Why might gene expression on the X be relatively tissue-specific? One reason for the low mean breadth of expression on the X could be a net influx of tissue-specific retroposed genes. However, the lower breadth of expression of genes on the X is only minimally accounted for by the accumulation of tissue-specific retroposed genes. When all-single exon (putatively retroposed) genes are removed, the global difference in the breadth of expression between autosomes and the X chromosome persists (0.28 versus 0.21, *p-*value < 2.2e-16). Moreover, the effect is not accounted for by tandem duplications on the X (with resulting narrowly expressed paralogs being counted more than once). To verify this, we performed an alternative version of the breadth of expression analysis. In the first step, we calculated the average breadth of expression for each gene family on each chromosome (that is for all family members on a given chromosome). Then the unweighted average of these averages was taken on the X and autosomes (ensuring equal contribution to the final chromosomal mean from each gene family regardless of its size). This analysis variant is not affected by tandem duplications as each gene family is given an equal weight in the final result. The difference in the breadth of expression between autosomes and the X persisted in this analysis (S14 Table) with BoE_autosomal_ = 0.35 versus BoE_X_ = 0.26 (*p* = 2.124e-10, Wilcoxon rank sum test with continuity correction).

One might alternatively suppose that if, after duplications, X-linked genes subfunctionalized more with regards to the tissue of expression, that this might explain a superficially lower expression breadth. One might imagine, for example, all gene families on autosomes and the X being expressed in the same number of tissues; but with higher subfunctionalization rates the X-linked genes might have a lower individual average expression breadth (but the same sum total expression breadth per family). However, this also appears not to be the case. In an alternative analysis, before the breadth of expression was calculated, expression levels were first summed for all transcripts mapped to a family on either autosomes or the X to provide a single sum total estimate of expression breadth per family. In this control, we find that autosomal family members still have a higher breadth of expression than do X-linked paralogs (S14 Table; 0.69 versus 0.43, *p* < 2e-16, Wilcoxon rank sum test).

As the X chromosome is inactivated in the germline of males, which have the XY genome, the germline inactivation hypothesis ([15,16], see also [17]) suggests that genes involved in spermatogenesis were transferred away from the X. As a consequence, any housekeeping gene with germline expression would need to be relocated from the X, or have its germline expression somehow compensated. However, the avoidance of germline expression alone cannot explain the reduced breadth of expression on the X; there are only two testicular libraries (and testes are mixtures of cell types) and the breadth of expression calculated in somatic tissues alone is still highly biased (Table 7). Similarly, excluding from the analysis any genes expressed in testes or in meiosis does not affect the conclusion that X-linked genes have a lower maximal expression and a lower breadth of expression (S15 Table). The germline inactivation hypothesis also could not explain the existence of the cap on maximal expression on the X and its enrichment of testis-specific genes (Fig 3). The metric of binary exclusion from the X suggests that germline expressed genes are not preferentially excluded (S1*A* Fig and S10 Table).

**Table 7:** The ratios of autosomal-to-chromosome-X breadth of expression (BoE_autosomal_/BoE_X_) for selected subsets of tissue samples (for example, male-specific, female-specific, brain-specific, *etc*.).

Tissue subset	Samples included	BoE_autosomal_/BoE_x_ (*100%)	Statistical significance	Interpretation
All	All FANTOM5 tissues	142%	*p* = 1	BoE is higher on autosomes than on the X
			*p*_X_ = 1	
			*p*_autosomal_ = 1	
Male-specific tissues	Epididymis, penis, prostate, seminal vesicle, testis	146%	*p* = 5.186499e-112	The above effect is much weaker in female tissues, and stronger in male tissues
			*p*_X_ = 2.605337e-15	
			*p*_autosomal_ = 2.533286e-100	
Female-specific tissues	Breast, cervix, ovary, uterus, vagina	133%	*p* = 1.233069e-60	
			*p*_X_ = 5.346621e-08	
			*p*_autosomal_ = 1.154556e-53	
Non-sex-specific tissues	All tissues except for male and female- specific tissues	142%	*p* = 0.04529611	No impact
			*p*_X_ = 0.2716826	
			*p*_autosomal_ = 0.06438631	
Brain tissues	All 75 FANTOM5 brain libraries	134%	*p* = 1.326906e-38	The effect is much weaker in brain tissues (especially those of fetal origin)
			*p*_X_ = 0.0004761647	
			*p*_autosomal_ = 8.973315e-36	
Adult brain tissues	All 75 FANTOM5 brain libraries excluding fetal and newborn	134%	*p* = 2.687509e-33	
			*p*_X_ = 5.255054e-05	
			*p*_autosomal_ = 7.152857e-30	
Fetal brain tissues	Fetal brain, occipital lobe, parietal lobe	130%	*p* = 0	
			*p*_X_ = 3.265555e-34	
			*p*_autosomal_ = 0	
Newborn brain tissues	Newborn caudate nucleus, cerebellum, globus pallidus, hippocampus, medial frontal gyrus, medial temporal gyrus, occipital cortex, occipital cortex, parietal lobe	136%	*p* = 3.99421e-230	
			*p*_X_ = 1.470615e-23	
			*p*_autosomal_ = 1.597182e-210	
Germ-line	Testis, ovary	142%	*p* = 5.897246e-66	No impact
			*p*_X_ = 8.241411e-13	
			*p*_autosomal_ = 3.194485e-56	

NOTE: Here, we grouped tissues in selected subsets most relevant to X biology (unique to male or female, sexual versus nonsexual, generative versus somatic, etc.). Lower values of BoE_autosomal_/BoE_X_ suggest relatively higher expression on the X. From Table 7, it is clear that the general trend for BoE to be higher on autosomes than on the X chromosome holds for all these tissue subsets. However, the effect was less marked in female tissues.

A possible sexually antagonistic explanation for the reduced breadth of expression on the X is that selection towards sex specialization, if occurring at an extreme level for the majority of X-linked genes, might reduce the global breadth of expression on the X. Imagine a gene expressed in many tissues including, let us say, prostate (which is a male-specific somatic exocrine gland). Imagine now a mutation in a broadly expressed gene that makes for a better functioning prostate, but at the cost of a reduced performance in females. Following Rice’s hypothesis, it is possible for such a mutation to spread. The deleterious effects in females can be mitigated by reducing female expression. The net effect might be male, possibly prostate-specific, functions. If so, the trend to sex-specificity might explain a trend to the lower breadth of expression. This model predicts that the loss of expression in non–sex-specific tissues is responsible for the overall decrease in the breadth of expression on the X. To control for this, we performed an alternative analysis in which the breadth of expression was measured only in tissues that are not sex-specific (that is, excluding the epididymis, the penis, the prostate, the seminal vesicle, the testis, the breast, the cervix, the ovary, the uterus, and the vagina). We found no impact: BoE_autosomal_/BoE_X_ was still equal to 1.42 (Table 7). Moreover, as noted above, the analysis of binary exclusion from the X (S1*A* Fig and S10–S12 Tables) suggests that all tissues except substantia nigra are statistically significantly excluded from the X, consistent with a general non-tissue-specific move away from the high breadth of expression (*p-*values in S10–S12 Tables are calculated by Fisher’s exact test, see Methods for the definition of binary exclusion). Thus, sex-specific tissues were not extreme outliers to the general trend for the exclusion (which conflicts with the hypothesis of sex specialization on the X).

The breadth of expression on the Y, the X, and autosomes runs in the same order as the inverse of the recombination rate; the non-recombining Y has the lowest breadth of expression (Table 1), the more highly recombining autosomes have the highest breadth of expression (the X chromosome being intermediate). A mechanistic coupling between reduced recombination and the reduced breadth of expression is easy to envisage. A reduced recombination rate could result in a weakened purifying selection or a reduction in GC-biased gene conversion. As transcription factor binding sites are known to be GC-rich [15], it is possible that the loss of recombination on the Y and the X thus resulted in the loss of transcription factor binding sites by either accumulation of deleterious mutations, or reduced levels of biased gene conversion and lower GC-content. That mean isochore and promoter GC-content of the three chromosome classes also run in inverse relation to the recombination rate lends credence to such models.

However, several lines of evidence argue against this hypothesis as the full explanation. First, whilst the rate of recombination correlates positively with exonic GC-content at third sites (GC3) and mean isochore GC-content, it does not positively correlate with promoter-CpG, promoter GC-content, TfbsNo, or the breadth of expression (Table 8). This suggests that GCs of functional promoter elements are resistant to weakened selection or biased gene conversion. Moreover, we might have expected that domains that have had reduced recombination rates for longer time spans would have shown more evidence of apparent decay, but this is not the case. The distribution of the breadth of expression along the X chromosome does not fit well with the strata structure on the X (S5 Fig). Indeed, the breadth of expression profile is fairly uniform in different strata along the X chromosome (S5 Fig). Despite this, for 60 transcripts on strata 8–12 (as defined in [47]) there is evidence for an increased proportion of tissue-specific expression (S2 Fig and S5 Fig). Genes within strata 8–12 were exclusively tissue-specific and there are no housekeeping transcripts in this cluster. These transcripts were on average expressed narrowly (the mean breadth of expression on the X, strata 8–12: BoE_Xstrata8-12_ = 0.059) but in a variety of tissues, with the top ten being pineal gland, heart, breast, small intestine, ovary, colon, uterus, throat, placenta, and adipose. Removal of strata 8–12 from the analysis does not affect the conclusion that X-linked genes have a lower breadth of expression (BoE_autosomal_ = 0.3, the mean breadth of expression on the X, strata 1–7: BoE_Xstrata1-7_ = 0.22, *p* < 2.2e-16, a Wilcoxon test).

**Table 8:** Correlations with the recombination rate.

Variable	Spearman correlation with the local recombination rate	
Isochore GC-content	*p* = 8.411e-07	*rho* = 0.13 (*)
GC3	*p* = 2.074e-08	*rho* = 0.15 (*)
Promoter GC-content	*p* = 0.12	*rho* = 0.04
CpG	*p* = 0.4229	*rho* = -0.02
TfbsNo	*p* = 0.0002427	*rho* = -0.096 (*)
The breadth of expression	*p* = 0.1755	*rho* = 0.036

* Signifies a statistically significant correlation.

NOTE: A genetic map of the recombination rate (in cM/Mb) mapped onto nucleotide positions for the X chromosome was obtained from HapMap II [48,49]. The map reports the recombination rate at the average resolution of 1,699 base pairs (bps), which is comparable to the proximal promoter size.

Can the reduced GC-content explain the reduced breadth of expression on the X? One should note that the breadth of expression does not depend strongly on isochore GC-content (S6 *E* Fig); instead it co-varies with a promoter’s GC-content (S6*B* Fig). Moreover, even if we compare X-linked genes with autosomal genes of matched promoter GC-content, we see that the X still has a reduced breadth of expression (S7*C* Fig). Considering either proximal promoter GC-content (S6*A*–S6*C* Fig) or isochore GC-content (S6*D*–S6*F* Fig), we construct a *loess* regression coupling GC-content of either the proximal promoter or the surrounding sequence and the breadth of expression. Considering the residuals from this regression (S6*C* and S6*F* Fig), we see the X to have a much reduced breadth of expression controlling for GC-content, the same not being seen on the Y chromosome (although sample sizes are more limited here). We conclude that a reduced breadth of expression on the X cannot be accounted for solely in terms of reduced GC-content associated either with mutational decay or with reduced rates of biased gene conversion.

#### The low breadth of expression on the X is explained by the limit on maximal expression

The above results suggests that the lower breadth of expression of genes on the X is robust, not owing to biased gene content, and not explained by either the germline inactivation/SAXI model, nor the sexual antagonism model, nor the reduced recombination model. Might the limit on maximal expression explain the finding? If expression at high levels suddenly becomes impossible on a chromosome then this is most likely to affect broadly expressed genes. This is because the chance of having an intolerable maximal expression level in at least one tissue is higher the more tissues the gene is expressed in (as there are more opportunities to “fail”). To see this, consider two genes, one expressed in ten tissues and one expressed in just one. Let us assume that for both the genes the expression level in each tissue within which they are expressed is drawn at random from the same underlying distribution. This being so, the maximum for the broadly expressed gene is very commonly going to be higher than the maximum for the tissue-specific gene. For simplicity we can rank order 1–11 (1 being the highest) the expression levels of our two genes in 11 conditions in which they are expressed. Only one in 11 times would the tissue-specific gene be granted the value 1 (the highest expression level). In 10 out of 11 times, the higher maximal expression would be granted to the broadly expressed gene. Thus, as the proto-X evolves to the X we expect broadly expressed genes to come under selection to divest functions to autosomes (or otherwise reduce expression) thereby reducing breadth. The ones left on the X will have then lower than the average maximal expression and a lower breadth of expression.

From the sampling effect noted above, we expect a correlation between the maximal expression level and the breadth of expression. This is not to say that there might not in addition be interesting biology to explain any such correlation, just that such a correlation does not a priori demand further rationale. As predicted, there is a strong correlation between the breadth of expression and maximal expression (*rho* = 0.78, *p* < 2.2e-16 for all genes; *rho* = 0.55, *p* < 2.2e-16 for housekeeping genes—defined as those with the breadth of expression higher than 0.66). More anecdotally, the 159 autosomal genes with the very highest expression had a breadth of expression 56% higher than background genes (the breadth of expression of 0.46 versus 0.295, *p* < 2.2e-16).

**Fig 7:**
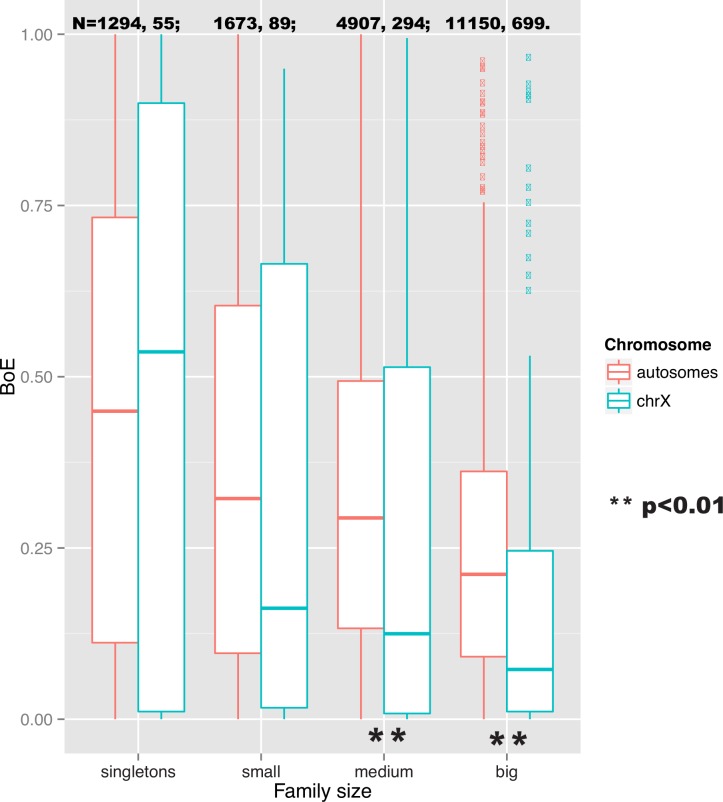
A shift towards a lower breadth of expression on the X is duplication-dependent. This figure shows boxplots for the breadth of expression depending on the chromosomal location and gene family size. Only for medium (more than two members) and big gene families (more than five members) is there a difference in the breadth of expression between autosomes and the X, suggesting the effect is duplication-dependent. The underlying data can be found at http://fantom.gsc.riken.jp/5/data/ and in Dryad Digital Repository (doi:10.5061/dryad.p4s57).

If divestment of high expression of some broadly expressed genes to autosomal paralogs is seen, might singleton genes be different? Intriguingly, the analysis of the breadth of expression depending on the chromosomal location and the size of a gene family, suggests the effect of a lower breadth of expression on the X is duplication-dependent, as it is not observed for singleton genes (Fig 7). In other words, the shift in the breadth of expression between autosomes and the X could be facilitated by the presence of pre-existing paralogs, or the ability to generate new ones after the X was formed. Paralog-based compensation of the reduced expression level would not have been possible for singletons so perhaps they, instead, found alternative means to increase their expression or tolerated reduced levels.

The maximal expression limit model can thus explain why X-linked genes have, on average, a relatively lower breadth of expression than autosomal genes. This is because the functions of X-linked genes demanding high expression in any given tissue would need to be divested to autosomes or otherwise be lost. The same model would also, however, predict that housekeeping genes with low maximal expression in all tissues could be tolerated on the X and hence that there is no avoidance of broad expression per se. Similarly, we expect very highly expressed but tissue-specific genes also not to be tolerated on the X (Table 2). Consistent with the former, highly broadly expressed genes remaining on the X have lower average maximal expression than broadly expressed genes on autosomes (maximum_autosomal_ = 483 TPM, maximum_X_ = 346 TPM, when the breadth of expression lower than 0.85 for both X and autosomal genes, *p* = 0.004509 in a Wilcoxon test). This suggests that X-linked genes retain broad expression profile only if they had, or can evolve, relatively low level expression in all tissues.

We hypothesize that the haploid expression of genes on the X in the heterogametic sex is the core issue, the problem being exacerbated by X inactivation in females. If this were so, then we would expect a reduced breadth of expression in birds as well, as the Z-chromosome has the same problems in female birds as the X does in male mammals. In line with this expectation, as noted above, we indeed find that Z-linked genes in birds also have a lower breadth of expression and maximal expression than autosomal genes, although to a much smaller extent than seen in mammals. These results (BoE_Z_ = 0.138 versus BoE_autosomal_ = 0.16, Wilcox *p* = 0.04334; average Maximum_z_ = 291 versus Maximum_autosomal_ = 341, *p* = 0.026; absolute Maximum_z_ = 21,476 and absolute Maximum_autosomal_ = 145,465) suggest the trend to be repeatable (and not reflecting some accidental bias in the genes on the proto-X prior to X’s formation).

### Highly Expressed Autosomal Genes Seeded Promoter-less Retro-Copies on the X, Giving Rise to Tissue-Specific Genes with Low Maximal Expression

The analysis of retroposed genes provides further tests of the traffic jam hypothesis. Just as genes ancestrally highly expressed on the proto-X cannot, we suggest, sustain themselves on the new haploid-X, so, too, retrocopies from highly expressed autosomal genes on to the X chromosome should be weakly and narrowly expressed. As reported previously [50], a high proportion of paralogs on the X were retroposed from autosomes (in our data this proportion was 3.9%, please see Table 5 and Table 6). We find that a retroposition to the X was accompanied by a greater reduction in the breadth of expression (in comparison to the autosomal parental gene), maximal expression, and TfbsNo than that observed for non-retroposed autosomal-X paralog pairs (ΔTfbsNo = -10.42 for retroposed versus -3.91 for non-retroposed, consistent with the promoter-less mechanism). High maximal expression of autosomal paralogs of X-linked retrogenes (Table 5, *X→autosomal* pairs) suggested a scenario where highly expressed autosomal genes, many of which were transcription factors, seeded retro-copies on the X that are much more tissue-specific and weakly expressed than their parental genes (with on average a 22-times lower maximal expression level, see Table 5).

Perhaps most striking is the finding that, as predicted, autosome-to-X retroposition events are associated with lower maximal expression of retrogenes on the X (the mean is 32±61, where “±” signifies standard deviation), than seen for X-to-autosome retrogenes (the mean of 129±293, *p-*value = 6.347e-12 in a Wilcoxon test). This test controls for the mode of duplication and so is perhaps the clearest indication of the importance of being on the X chromosome as regards to a low maximal expression level.

Both retroposed and non-retroposed X chromosome paralogs diverged asymmetrically after gene duplications, with the X-linked paralog being more tissue-specific and having fewer transcription factor binding sites (Table 5 and Table 6). After the X was formed, two waves of gene duplications facilitated remodeling of its content towards tissue-specific expression: Eutherian (with approximately 50% retropositions) and Therian (with only 9% retropositions). However, older pre-existing genes, derived from two rounds of whole genome duplication (2R-WGD), chordate and bilaterian duplications also experienced pressure to exclude housekeeping genes and genes with high maximal expression from the X. 2R-ohnologs (2ROs) represented the most numerous wave of duplications in the human genome [51] and the X chromosome was no exception (with 572 out of 1,078 X-linked duplication nodes mapping to 2R-WGD, see Table 6).

### Some Evidence That the X Chromosome Is Adapted to Low Maximal Expression

Assuming the maximal expression level to be the key issue, one might also expect the more highly expressed X-linked genes to have evolved some other adaptations to enable higher transcription or translation rates in the face of haploid expression. X-linked genes could have evolved longer half-lives of their mRNAs or proteins. Alternatively, mRNAs of X-linked genes could be more capable of ribosome binding than equally expressed autosomal genes, enabling more protein production per transcriptional event. Some of these issues have recently been analyzed and indeed, X-linked genes have longer mRNA half-lives and a higher density of ribosomes [52]. This is consistent with X-linked genes being adapted to making the most of their relatively low expression levels.

Two further possible adaptations that we can examine are alternative transcriptional start sites (TSSes) and tandem duplications. Both of these provide possible mimics to the diploid situation by doubling the number of promoters available for transcription factor binding. We find no evidence to support the former possibility. The average number of TSSes per gene on autosomes is, if anything, higher than on the X chromosome (4.41 versus 3.95, Wilcox one-sided test *p* = 0.0263).

The formation of tandem duplicates would result in larger gene families, but genes on the X tend to belong to smaller families than genes on autosomes (an average X-linked gene belongs to a family of 2.72 genes, while an average autosomal gene belongs to a family of 5.76 genes, Wilcox *p* < 2.2e-16). This, however, does not address the core issue, namely whether X-linked genes duplicate more post the formation of the X chromosome. Consistent with the adaptation to traffic jam model, duplicability since the formation of the X (see Methods for the definition of duplicability) is twice as high on the X in comparison to autosomes (1.11 versus 0.55, *p-*value < 2.2e-16 in a Wilcoxon test). Moreover, the X has only 22% (166) singleton genes versus 39% (7,411) on autosomes. However, we might also expect the selection for duplicate retention to be the strongest on the more highly expressed genes, but we see no evidence for higher duplicability for high maximally expressed genes on the X. In fact, there is an overall negative correlation between duplicability and maximal expression (*rho* = -0.22; *p-*value < 2.2e-16 on autosomes; and *rho* = -0.34; *p-*value < 2.2e-16 on the X chromosome), most simply explained as a duplication bias towards non-essential/lowly expressed genes [53], seen in other taxa. Similarly, when we divided X’s non-singleton genes into high and low maximally expressed using the median maximal expression (that is 31 TPM) as the cutoff and then calculated average duplicability, the result for low-maximal genes was 1.67 and for high-maximal only 0.57 (*p* = 3.028e-10 in a Wilcoxon test).

One might however argue that if a highly expressed X-linked gene had duplicated in order to increase its total net dosage, then those that had not duplicated should have the highest expression per gene. The duplicates could have lower expression per gene, but a higher net expression when adding together the contribution from each duplicate. Arguing against this, however, is the finding that for many gene families (e.g., MAGE) duplicates tend to be specialists for expression in different tissues, so the net expression in any one tissue is approximately the expression of the most highly expressed paralog in that tissue. This suggests the net expression level is not the driver of duplications and the trends are better explained as a bias towards the retention of genes that are less likely to be deleterious immediately after the duplication.

## Discussion

Above, we have provided much evidence suggesting that an important force shaping gene expression on the human X chromosome is nothing as nuanced as sexually antagonistic variation nor the avoidance of germline X inactivation, but rather might be a simple incapacity of haploid-X-linked genes to be expressed at very high rates. In particular, we have observed that X-linked genes have lower maximal expression than autosomal genes and that highly expressed X-linked genes appear to be less able to increase their expression than lowly expressed X-linked genes and than autosomal genes. That a lower maximal expression is seen for autosomal-to-X retrogenes than for X-to-autosomal retrogenes is consistent with these trends. The limit to maximal expression levels on the X can also explain many of the trends regarding the sorts of genes preferred and avoided on the X and the lower breadth of expression of X-linked genes. That highly expressed tissue-specific genes are also avoided on the X, while lowly expressed but broadly expressed genes are not avoided, suggests that a maximal expression level, rather than the breadth of expression per se is the issue at stake, the breadth effect resulting from a tendency for broadly expressed genes to have a high maximal expression level in at least one tissue higher than is tolerable.

Our results accord with what is seen in the fly testes. In this structure, there is an absence of dosage compensation (as in mammals) suggesting that the same traffic jam as seen on the mammalian X might exist on the fly X. Meiklejohn and colleagues [23,24] have indeed demonstrated that the paucity of X-linked male-biased genes in *Drosophila* is driven primarily by the lack of dosage compensation of the X in the testes, implying that the haploid dose of the fly’s X without dosage compensation has a maximal expression level lower than the diploid X in females. As we noted in the introduction, the fly X tends to be hypertranscribed in other tissues and so is not so restrictive to genes with high maximal expression, but is likely to constrain the evolution of even higher expression level.

The traffic jam hypothesis highlights issues that might be worthy of future scrutiny. For example, Pessia et al. [34] noted that certain classes of X-linked genes involved in protein–protein interactions were up-regulated on the mammalian X. These corresponded to particularly large protein complexes. If up-regulation is difficult on the X, how do some genes manage to achieve it? Were those genes lower in expression to begin with? Similarly, we see an increase in expression since the human/Chimpanzee ancestor for testis-expressed genes [54]. How mechanistically did this happen, given the overall difficulty in increasing expression on the X?

More generally, we can ask what exactly happened as the proto-X became the X. A change in the breadth of expression appears to be particular to genes with autosomal duplicates. Might this be because X-linked genes with autosomal paralogs redirected their transcription factors to the promoters of autosomal copies, effectively automating a switch in expression from the X to autosomes with no further adaptation? We term this scenario the physiological model, as it requires no selection. Alternatively, might the creation of new autosomal paralogs have been central to an evolutionary, rather than simply physiological, adjustment? In principle, one can ask whether X-autosomal paralogs increased autosomal expression if the pair existed prior to the formation of the X, by comparing the maximal expression levels to those seen in chicken. Unfortunately, after rigorous filtering, this test leaves too few human-chicken tetrads (namely 20) to be informative.

An alternative analysis is to compare age-matched autosomal-autosomal versus autosomal-X paralogs, split between those whose paralogs formed before or after X’s formation. The general expectation is that, if the lower breadth of expression on the X is compensated by increased expression on autosomes, autosomal paralogs of X-linked genes should have a higher breadth of expression than autosomal-autosomal paralogs of matched age. Indeed, we find some evidence for this. We find an elevated breadth of expression and an elevated maximal expression of autosomal paralogs of X-linked genes, but only for newly formed paralogs (i.e., those dating to the taxon Theria or younger) and not for duplicates pre-dating the formation of the X (S8 Fig). Thus, this analysis provides no evidence for the hypothesis of the immediate physiological response, but supports the notion of an active evolutionary divestment from the X to autosomes.

Despite the apparent power of the traffic jam hypothesis to explain many curiosities of X’s gene content and evolution, we do not wish to suggest that the reduced maximal expression level necessarily explains all curious features of gene expression on the X. For example, as we noted in the introduction, the GC-content on the X is most likely a consequence of reduced recombination. Indeed, we observe that the haploid-expressed X and Y differ in several regards. For example, there is an even more extreme poverty of transcription factor binding sites on the Y (Fig 5). Using our standard analysis conditions (that is the quality cutoff of 500 and the window size of 1 kb), we detected only 2.88 transcription factor binding sites per a RefSeq gene on the Y (compare with values for the X and autosomes given in S13 Table). As both the X and the Y are haploid-expressed these differences in TfbsNo between the X and the Y suggest other forces are at play. Two explanations are most evident, these being that Y-linked genes could not sustain transcription factor binding sites for expression needed in females, or decay associated with the lack of recombination/GC-biased gene conversion.

### A Relationship between Maximal Expression Constraint and the Germline X Inactivation Model

While by no means being a full explanation, the possible centrality of the upper limit on the expression level on the X chromosome calls into question evidence in support of other models. For example, it was noted in support of the X inactivation model that many X-linked genes spawned autosomal retrogenes with high expression levels in male germline [19]. However, as previously noted [19], the model makes only a partial explanation of the facts, as many of those germline expressed genes are highly expressed at the times when the X chromosome is not inactivated. Our model places emphasis on the highly expressed issue more than it does on the germline expression issue. The selection we suggest may be to enable high expression from a chromosomal environment incompatible with high-level expression in any tissue (not just the germline). Indeed, we suggest that the logical order of events likely requires a gene movement prior to the evolution of X inactivation (as noted by the SAXI model [18]). If one considers the process in the other direction then the explanation appears unparsimonious; if the X has shut down and essential male germline genes have not moved away, then the shutdown of the X might well lead to sterility. Thus, only if the essential genes already have moved from the X could X’s inactivation have been favored. But if the movement off the X was prior to X inactivation, then X inactivation could not have caused the movement.

If this logic, as previously voiced [18], is correct, this suggests that germline X inactivation is a follow-on consequence enabled by, and not the cause of, the movement. Why then might male germline be especially involved? The SAXI [18] hypothesis suggests sexual antagonism as being at the heart of the issue. The emphasis on retrogenes here possibly provides an alternative answer. By definition, for retrogenes to be created and transmitted requires germline expression. If male germline expression is higher than female germline expression, then we expect many highly expressed male germline genes to be favored to spawn autosomal copies (for no better reason than because it enables higher expression in the germline following the formation of the haploid-expressed X chromosome). In addition, the chance of retroposition will be the highest for those genes that are most highly expressed on the X (more RNA transcripts), thus rendering the movement away from the X not only advantageous (for dosage reasons) but also more likely for highly expressed germline expressed X-linked genes. In principle, then, the loss of testes-expressed genes to autosomes via retroposition may be selectively advantageous without having to evoke sexual antagonism. The loss of the expression of an X-linked paralog may happen before or after X inactivation.

### Evidence Supports Reproduction-Specific Rather Than Sex-Specific Gene Enrichment on the X, Questioning the Model of Sexual Antagonism

That we see an equally reduced breadth of expression for X-linked genes when we remove sex-specific tissues from the analysis suggests that the maximal expression argument has a much broader explanatory power than the sexually antagonistic model, although it is hard to discount sex-specific selection on alternative alleles in non sex-specific tissues. But might the facts that appear to support the sexually antagonistic model actually be alternatively understood in the context of a limit on X-linked gene expression levels?

Such a reinterpretation has been defended for flies. Vicoso and Charlesworth [26] consider in the case of the fly that a direct coupling between sex-biased expression and expression possibilities on the X might be limited. The X is hyper-expressed in the fly so, they suggest, increasing expression further on the X is likely to be improbable. As male-biased expression predominantly occurs via up-regulation, a cap on expression level they suggest might thus explain the dearth of genes biased towards male-specific expression on the X. Incidentally, it is not transparent as to why the fly X evolved hyper-expression while the mammalian X apparently did not. The proportion of all genes on the fly X is much higher than in mammals and birds and so the net selective effect of not hyperactivating may be lesser on the mammalian X, but this is just a speculation. It is also not clear why testes-expressed genes can increase the expression level on the human X [54], when most other genes cannot.

While the X appears not to be hyperactivated in mammals, a difficulty in up-regulating genes on the X may be an issue (see evidence above). Our data, however, suggest that expression in testes is especially common on the X chromosome, in contrast to what is seen in flies. Is then our data broadly consistent with the sexual antagonism model as an additional force, beyond the maximal expression limit? Evidence looks supportive at first glance. We find that genes with specific expression in many sex-specific tissues show enrichment on the X (the epididymis, the testis, the uterus, see Fig 3). Although maximal expression levels of these tissues’ tissue-specific genes is low (in part accounting for over-abundance), this enrichment appears to be greater than expected even allowing for decreased maximal expression levels of genes in these tissues (Fig 3).

While at first glance this is broadly consistent with the sexually antagonistic model, for reasons unknown, amongst the set of tissues enriched in X-linked expression is the placenta (also enriched after controlling for its relatively low TSME). The placenta is especially noteworthy as it is not sex-specific but reproduction-specific. This questions whether prior trends ascribed to sexual antagonism might not have an alternative explanation, possibly related to the commonly reported faster evolution of reproduction-related genes [55–57] and hemizygosity of the X. Against such a hypothesis is the recent finding that in mammals any faster sequence evolution of X-linked genes is not owing to hemizygosity, but can be accounted for in terms of GC-bias and low expression [58]. No matter what the explanation, that the trends are seen after controlling for expression maxima suggests that the enrichment of reproduction-related genes is not simply explained by our maximal expression hypothesis, but neither is it necessarily owing to sex specificity per se.

### The Enrichment of Brain Expression on the X May Predate the X

It is commonly the case that patterns of enrichment of certain functional classes of genes on the X prompt adaptive speculation. Above we caution that fixation on the problem of sex-specificity may have distracted us from the general issue of reproduction-specificity. A more general caveat concerns whether an adaptive explanation is needed at all. The other tissue whose preferentially expressed genes are over-represented on the X, allowing for maximal expression, is the brain (see analog enrichment data in Fig 4 and S3 Table and binary enrichment data in S4–S6 Tables). Brain tissues are also among the least depleted in expression on the X using the metric of binary exclusion (S1*A* Fig, S10–S12 Tables, Wilcoxon one-sided test *p* = 0.05994 when brain tissues are compared against non-brain tissues). The last effect holds even after controlling for brain’s relatively low TSME (Fig 3).

Although the enrichment of brain-specific genes and links with mental-retardation disorders on the X were previously known [59,60], FANTOM’s broad collection of brain-related libraries allows a new appraisal of the extent of this trend, with most X-linked brain-specific genes being expressed widely in the hindbrain, the midbrain, the brain stem, as well as in libraries from adult, newborn, and embryonic brain samples. Using the binary enrichment metric (see Methods), almost all brain tissues (the brain stem, the midbrain, the higher brain, as well as brain glands such as the pituitary) were relatively highly expressed on the X (S3 Table). On the cellular level, we found that brain X-linked genes were enriched in astrocytes, perineural cells, neural stem cells, and neurons (S5 Table) underlining the broad functionality of these molecules across the nervous system.

How to interpret an excess of brain-expressed genes on the X, which exists before and persists after controlling for maximal expression (Fig 3), is uncertain. We suggest that caution is advisable in ascribing a selectionist explanation, as the association with brain appears to predate the formation of the proto-X. For example, X-linked brain-specific expression is associated with duplications predating the formation of the X (taxa Amniota and older, S16 Table). Similarly, there are numerous examples of X-linked brain-specific genes that exist on autosomal syntenic regions in chicken (see S9 Fig for the map of human-chicken orthology) and are brain-expressed in chicken (see S17 Table for four examples).

For the most part, then, it is unresolved whether the brain enrichment on the X chromosome reflects an accident of history or whether the retention and later evolution require a special explanation. We note, in favor of the latter, that the MAGE family of tumor-testis-specific antigens has undergone much duplication and tends to be expressed across the brain (and frequently also in reproductive tissues, either male, or female, see S18 Table).

### Conclusions

Recent evidence suggests that, unlike the fly X chromosome, the mammalian X is not hyper-expressed. Rather, it is expressed at levels approximately equivalent to those of the ancestral autosome (per promoter). As such, we might expect the X not to be a tolerable environment for genes with very high maximal levels of expression (as all the expression, in males at least, must run through just a single promoter). Moreover, we might expect that genes relatively highly expressed on the X would be constrained in their ability to increase their expression levels. The analysis of expression data supports all of these predictions. The traffic jam model also explains the known fact of the lower breadth of expression for genes on the X (and the bird Z), as genes with broad expression are, on average, those with high maximal expression. The traffic jam model also predicts that tissues in which tissue-specific genes are very highly expressed (e.g., secretory tissues, tissues abundant in structural proteins) are also tissues in which gene expression is relatively rare on the X. These trends cannot be fully accounted for in terms of alternative models of biased expression. These results suggest that a force no more subtle than limited rates of expression on haploid chromosomes is a fundamental driver of the biology of the mammalian X chromosome.

## Methods

### RefSeq Transcripts and Inferred TSSes

There were 44,218 human RefSeq transcripts. This total consisted of 36,382 mature messenger RNAs (NM-accessions), and 7,836 non-coding transcripts (NR-accessions). The non-coding transcripts included structural RNAs and transcribed pseudogenes (no filtering was performed on the dataset). At the beginning of the analysis, RefSeq transcripts (mapped onto February 2009 human genome assembly—hg19) were downloaded from the UCSC Genome Browser (http://genome.ucsc.edu/) in the Browser Extensible Data (BED) file format, using the table browser tool (http://genome.ucsc.edu/cgi-bin/hgTables). The beginnings of RefSeq transcripts (that is the start positions of mapped transcripts) defined the location of TSSes.

### ENCODE Data

ENCODE ChIP-seq data define the location of transcription factor binding sites and were processed as previously described [46]. To be specific, we used multi-cell-line clustered data, from the January 2011, data-freeze [19]. These data included 2,750,490 peaks for 148 transcription factors, derived from 71 cell-types with 24 additional experimental cell culture conditions [9]. The data were generated by eight different labs, adhering to a common and standardized set of protocols and controls [12]. The labs involved were as follows: the Myers Lab at the HudsonAlpha Institute for Biotechnology; the labs of Michael Snyder, Mark Gerstein and Sherman Weismann at Yale University; the lab of Peggy Farnham at UC Davis; the labs of Kevin Struhl at Harvard, Kevin White at The University of Chicago, and Vishy Iyer at The University of Texas Austin. For analyses of proximal promoters, we used only the highest quality transcription factor binding sites, with the reliability score above five hundred points. However, as shown in S3*A–*S3*D* Fig, the trends discussed here were independent of, and robust to, the variation in the quality score. We also verified that results analogous to those for the January 2011, data-freeze (see S3 Fig) were obtained using a broader September 2012, data-freeze (ENCODE-TfbsV3 2012). The 2012 data-freeze consisted of 161 transcription factors and 91 human cell types under various treatment conditions [20].

To measure TfbsNo, ENCODE transcription factor binding sites were simply counted in a symmetrical window around the TSS, as previously described [46]. Unless stated otherwise, the size of the window was one kb (±500 base pairs) and only the most reliable ENCODE transcription factor binding sites (i.e., those with ENCODE quality scores above 500) were taken into account, which proved representative in previous analyses [46]). In previous work, we also explored various ways of measuring TfbsNo (that is simple counting, counting unique sites, and counting TfbsNo excluding PolII) and showed them to be essentially equivalent and all a good predictor of the breadth of expression [46].

S13 Table shows that TfbsNo is much lower on the X for all promoter window sizes and regardless of whether all transcription factor binding sites or only those with the ENCODE quality score above 500 were considered. As described previously [46], the distribution of transcription factor binding sites per gene was highly non-normal and followed a power law (S13 Table).

### The FANTOM5 Dataset

CAGE tags were mapped to RefSeq transcripts +/-500 base pairs (bps) from their TSSes and normalized to tags per million (TPM), as previously described [37,46]. The signal of ten TPM was chosen as the cutoff for a gene to be classified as “on” (this cutoff was accepted as the standard for human data throughout the consortium). FANTOM5 is the most comprehensive expression dataset ever generated, including 952 human and 396 mouse tissues, primary cells and cancer cell-lines. FANTOM5 is based on cap analysis of gene expression (CAGE) a unique technology that characterizes TSSes across the entire genome in an unbiased fashion and at a single-base resolution level [21]. CAGE automatically sums expression levels of all transcripts beginning at a given transcription start site.

### TreeFam Gene Families

TreeFam release eight [61,62] was used for data on gene families and to infer paralogy, as previously described [46,63]. For expression analysis, multiple transcripts derived from the same gene were assumed to share the same evolutionary history (i.e., the same pattern of past duplications). When expressly stated in Results, the paralogy dataset was pre-processed to report only the most recent duplication event for each gene (that is the taxon of the youngest duplication node from which a gene descends).

We detected duplication events using an algorithm aiming at the reconciliation of the gene tree with the species tree [64]. The taxon of duplication was assigned using the principles of phylogenetic timing, that is to say dating duplication events on the basis of the phylogenetic distribution in extant species (assuming a known species tree). This data was used as previously described and as previously duplications with species intersection support equaled zero were not taken into account [51].

### Gene Families Expanded on the X Chromosome

From the total set of human duplications, extracted from TreeFam as described previously [46,51,63], we isolated only those which were dated to taxa younger than Amniota (that is Human, Homo/Pan/Gorilla, Catarrhini, Eutheria, or Theria) and where both children genes were located on the X chromosome. This dataset comprised 112 duplication events grouped in 36 families. This approach aimed at the identification of gene families specifically expanded on the X after its formation (rather than families that already had multiple members on the proto-X at the time of its formation). In S18 Table, we show the families with at least three X-linked gene members. To estimate the expression pattern of each of these families, we averaged expression levels of their transcripts and reported the top five tissues of expression.

### GC-Content

The GC-content of the proximal promoter was calculated for the nucleotide sequence in one kilobase pair (kbp) around the TSS (±500 bps) on the hg19 human genome assembly. The *R* function *alphabetFrequency* from the package Biostrings was used. To calculate the GC-content of the isochore, a 20 kilo base pairs (kbps) window around the TSS was used (±10,000 bps). Unless stated otherwise, a masked genome sequence from the *R* package *BSgenome*.*Hsapiens*.*UCSC*.*hg19* was used with standard *BioC-Biostrings* functionality (masked for assembly gaps using RepeatMasker and Tandem Repeats Finder). Data points overlapping entirely with masked regions were omitted. To calculate GC3, coding regions were isolated from RefSeq sequences (according to annotations in the RefSeq GenBank file). Every third codon position was isolated into an *R* character vector and the *R* function *table* was used to calculate the frequency of GC nucleotides.

### What Is Meant by Maximal Expression, the Breadth of Expression (BoE), and Preferential Expression Measure (PEM)?

Maximal expression is the greatest numerical value attained (in TPM) for each transcript in any single library. Each library was processed separately: in a few cases where multiple donors were available for the same tissues, these were not averaged, to preserve information on the sex and age of the donor. Thus, the maximum does not arithmetically depend on the breadth of expression or average expression. Unless expressly stated otherwise, maximal expression was calculated for the human tissue set in FANTOM5. However, it should be noted that maximal expression correlated very highly between the three classes of human FANTOM5 samples. Maximal expression calculated for human tissues correlated with maximal expression for primary cells and cancer cell-lines with the values of *rho* of 0.71 (*p* < 2.2e-16) and 0.698 (*p* < 2.2e-16).

The breadth of expression was calculated per transcript, as the fraction of samples in which the transcript was detectable (using the cutoff of 10 TPM, as described previously [46]). Unless stated otherwise, the breadth of expression was calculated for the human tissue subset of FANTOM5. However, it should be noted that the breadth of expression correlated very highly between the three classes of human FANTOM5 samples. The breadth of expression calculated for human tissues correlated with those for primary cells and cancer cell-lines with *rhos* of 0.85 (*p* < 2.2e-16) and 0.84 (*p* < 2.2e-16), respectively.

Preferential expression measure (PEM) is calculated by taking the ratio of a normalized signal for a transcript in a library (in TPM) to the average signal of this transcript in all libraries of a given type. PEM was purposefully calculated for each CAGE library in relation to other libraries in its respective sample category only (that is either in tissues, or primary cells, or cancer cell-lines). In the case of brain, the presence of 75 libraries from different brain subsets (see S19 Table) could lead to the under-estimation of the brain PEM. We, therefore, verified that results analogous to Fig 3 could be obtained when PEM for each brain library is calculated only in relation to non-brain libraries (S10 Fig).

### A Randomization Control for Fig 1

We used a randomization procedure to estimate the probability that the skewed distribution of maximal expression on the X could be derived by chance. The total set of 31,095 transcripts was sampled one million times to pick a random subset of transcripts identical in size to the number of transcripts on the X (that is 1,433). After each sampling, the average maximal expression value was calculated for the sampling subset. *p-*value was estimated by counting the number of times the mean maximum of the sampling subset was lower than that observed for the entire data set (which was never, leading to *p* = 0).

### The Analysis of the Chicken Chromosome Z

FANTOM5 chicken libraries consisted of 25 CAGE libraries including: chicken aortic smooth muscles, hepatocytes, mesenchymal stem cells, leg buds, wing buds, embryo extra-embryonic tissue (day 7 and day 15), and whole body developmental time course (from 5 h 30 min to 20 d). The number of available data points to which TPM was normalized was limited by the number of annotated chicken RefSeq transcripts (which was approximately six times smaller than human, *n* = 4,426 on autosomes, and *n* = 241 on chromosome Z). Consequently, the cutoff for a gene to be classified as “on” was adjusted six times higher to 60 TPM.

### The Contribution of 2ROs to the Lower Breadth of Expression on the X

In the analysis of 2ROs, X-linked duplication nodes were defined as those with one autosomal and one X-linked paralog. Given their high frequency on the X chromosome, 2ROs can be estimated to account for $\frac{-120.12}{-208.3}\;=\;0.58$ of the overall reduction in the breadth of expression. This is calculated from data in Table 6 as the number of X-linked 2R-WGD duplication nodes times their shift in expression breadth, that is *N* * ΔBoE = −120.12, divided by the sum for all nodes and taxa, that is, $\sum_{i\;=\;human}^{bilateria}\;Ni\;*\Delta \text{BoEi}\;=-208.3$.

### Identifying Putative Retroposition Events

Single-exon genes were identified as putative retroposed genes. If the closest paralog to a putative retroposed gene had more than two exons, the pair was classified as a retroposition event. Further sub-classification was into either *X→auto*, *auto→X*, *auto-auto*, *X-X*, *X-Y*, or *auto-Y* retroposition events, depending on the location of the multi-exon parental gene. If both members of the paralog pair were single-exon, the retroposition was classified as non-directional.

### Functional Enrichment for Sets of Transcripts

DAVID version 6.7 (http://david.abcc.ncifcrf.gov/) was used for the enrichment analysis and clustering of gene annotations. DAVID works with the "DAVID gene concept," a method linking gene and protein identifiers across a number of databases such as National Centre for Biotechnology Information (NCBI), PIR and Uniprot/SwissProt [65,66].

### Understanding Tissue of Expression

For several analyses, specific subsets of FANTOM5 tissues were defined as follows:

1. Brain tissues, see S19 Table for the definition.
2. Constitutively male or female tissues are defined as those that must be of either male or female origin (i.e., which exist only in one of the sexes). Constitutively female tissues in FANTOM5 comprise of: uterus, vagina, breast, and ovary. Constitutively male tissues in FANTOM5 comprise of: epididymis, penis, prostate, testis, and seminal vesicle. Placenta has a unique status, being specific to reproduction rather than sex-specific (placenta is a mixture of maternal and newborn tissues; FANTOM5 placental RNA was purchased commercially and derived from dozens of pooled whole placentas collected at birth).
3. Facultative male or female tissues are those that could be either male or female, but in the FANTOM5 dataset were derived exclusively from one sex (S20 Table). Increased expression in these sample subsets would be indicative or either male or female preferential expression. However, we have found no evidence for this type of biased expression on the X chromosome.
4. Germ-line: testes and ovary. One should note, however, that germ-line expression is difficult to capture. Transcripts from bulk testis and ovary derive from multiple cell-types. Even expression profiling of isolated oocytes is not guaranteed to capture female germ-line expression as their transcripts derive from supporting and feeding cells.

### Calculating the Average Expression for the Top 1% or 0.1% Set of the Most Tissue-Specific Genes for Each Library

First, we selected the top set of 1% (that is 311 transcripts) or 0.1% (that is 31 transcripts) of the most tissue-specific transcripts (that is transcripts with the highest PEM) for each library. Then we calculated their average expression in a given tissue (by averaging signals obtained for each selected transcript, in TPM, in the tissue under consideration).

### Duplicability Since the Formation of the X

Duplicability was established as follows: for each non-singleton gene in the genome, the number of duplication events from which the gene was descending since the formation of X (taxa of duplication Human, Homo/Pan/Gorilla, Catarrhini, Eutheria, and Theria) was calculated. For example, if gene’s most recent duplication was at the base of vertebrates, duplicability equaled zero. But if gene’s most recent duplication is dated to taxon Theria, duplicability will equal one.

### Defining Enrichment Metrics: “Analog Enrichment,” “Binary Enrichment,” and “Binary Exclusion”

Three different ways of defining tissue-specific expression enrichment or exclusion on the X were explored, adding to the robustness of the analysis presented here.

Firstly, for each tissue we calculated the average expression (in TPM) of the X and autosomal genes (Fig 4), either for all or only tissue-specific transcripts (the breadth of expression <0.33), and calculated the ratio of average autosomal over the average X chromosome expression in a given tissue. We refer to this measure of differential expression as the metric of “analog enrichment” as it preserves the information on the level of expression of individual genes. A downside of this definition of expression enrichment is that the value of the metric could be dominated by a few very strongly expressed genes.

Secondly, we isolated the set of the top 1% most tissue-specific transcripts in each library and calculated the fold enrichment on the X for these transcripts against the random expectation based on the X-to-autosomal ratio of the total human gene set. *p-*values were calculated by Fisher’s exact test (S4 Table—human tissue, S5 Table—human primary cells, S6 Table—human cancer cell-lines; samples under-represented in expression on the X have the fold-enrichment values lower than one). We refer to this measure as the metric of “binary enrichment” in expression, as it is not affected by the strength of expression of individual tissue-specific genes.

Finally, we calculated the degree of “binary exclusion” from the X for each tissue. First, we classified all genes in a given tissue as either “on” or “off” based on the cut-off of 10 TPM (the standard definition used within the FANTOM5 consortium). Next, we compared the observed X-to-autosomal distribution of the “on” genes against the random expectation. The *p-*value was calculated by Fisher’s exact test. We refer to this measure as the metric of “binary exclusion” from the X. This metric is rather different from “analog enrichment” and “binary enrichment” outlined above, as instead of focusing on tissue-specific genes it defines the degree to which each tissue or cell-type contributed to the lower breadth of expression on the X (S10 Table—human tissues, S11 Table—human primary cells, and S12 Table—human cancer cell-lines).

### Z-Score Estimation

Brawand et al. [67] RNAseq gene expression data from six tissues in five primates was employed to estimate the extent to which any given human gene changed expression since the human-Chimpanzee common ancestor. Data in the file *NormalizedRPKM_ConstitutiveAlignedExons_Primate1to1Orthologues*.*txt* was employed with strand information from *Human_Ensembl57_TopHat_UniqueReads*.*txt*. Both files are in the supplementary materials of Brawand et al. [67]. This provides reads per kilobase per million mapped reads (RPKM) figures for 13,027 genes. We employed BayesTraits [68] to estimate the change in gene expression between current levels in humans and that seen in the human-Chimpanzee common ancestor. We employed the same phylogeny and branch lengths as those in Brawand et al. [67].

Brawand et al. [67] normalized RPKM values were passed to BayesTraits. For each gene, the mean of the normalized RPKM values in any given tissue in human was calculated separately for male and female samples. Similarly, under the circumstance that more than one male or female sample was available in any of the tissues in non-human primates, their mean was computed. If only one sample was described, this value was employed and pasted as an input to BayesTraits. To estimate the expression level for any given gene in the common ancestor of human and Chimpanzee, BayesTraits program was run to build the estimated gene expression tree for expression in males. This was done for each gene in each tissue. From the inspection of convergence trends, we concluded that the terminal 10% of BayesTraits estimates were robust. Given that this is not a point estimate but a series of estimates, we determined both the mean (*E*_a_) and variance (*V*_a_) of the estimated human-Chimpanzee ancestral state. We also examined the consequence of the relaxation the 10% cut-off and concluded that results were qualitatively unchanged.

The estimation procedure was implemented independently for each gene in each tissue. If *E*_current_ is the mean expression of a given gene, in a given tissue, in a given sex (or *E*_c_ in an abbreviated form), and the variance is *V*_c_, (assuming it to be estimable), while that for the ancestral condition is *E*_a_ and *V*_a_, then the degree of expression divergence we define as a Z-score:

$$Z\;=\;\frac{E_{c}-E_{a}}{\sqrt{V_{c}+V_{a}}}$$

Note that Z normalizes the extent of difference between the mean current expression level and ancestral level, by the variation both in current estimates (this could be expression noise or measurement error) and the magnitude of uncertainty in the human-Chimpanzee ancestral state. A Z-score greater than zero is indicative of an increase in gene expression since this ancestor. If we suppose that there is neither an increase nor a decrease in net transcriptional output in any given tissue, it might be reasonable to assume that for each tissue the median expression change must be zero. A minor adjustment of the *Z*-scores for all genes in all tissues is required to achieve this. If we designate the median Z-score (in any given tissue in a given sex) as *M*, then we can define modified Z as *Z*_mod_ = *Z*–*M*. After such a modification all tissues have a median Z of zero. All analyses were performed on *Z*_mod_. We refer to *Z*, for convenience, where *Z*_mod_ is what we are employing. Our method, note, has the advantage that it should be relatively insensitive to any RNAseq amplification biases (e.g., owing to GC-content): nucleotide content is almost identical between human and Chimpanzee and hence any amplification bias should affect human and Chimpanzee in equal measure. The degree of change from the ancestor, as assayed by Z, should then largely exclude amplification biases. As then expected, the mean correlation, across all tissues, between the change in GC (between human and Chimpanzee) and change in expression (i.e., Z-score) is indistinguishable from zero.

## Supporting Information

S1 Fig The degree of exclusion from the X chromosome for each sample. This figure consists of three panels marked as *a*–*c*. Data for human tissues (*a*), primary cells (*b*), and cancer cell-lines (c) are shown. Values above one on the *x*-axis signify exclusion from the X. The greater the degree of the exclusion, the greater the value on the *x*-axis. These charts are independent of the strength of expression of individual genes, as all data points were first converted into a binary (“on” or “off”). It is striking that gene expression in all samples is under-represented on the X by this measure (although brain tissues are least excluded). Details can be found in S10–S12 Tables.
(PDF)

S2 Fig A lower breadth of expression on the X is reflected in the dearth of housekeeping genes. This figure consists of four panels marked as *a* through *d* and showing density plots for the breadth of expression. Either all genes (*a* and *c*) or only-expressed genes are shown (*b* and *d*). In panels *c* and *d*, the breadth of expression is compared between autosomes and the entire X chromosome, while in panels *a* and *b* the breadth of expression is divided with respect to X’s strata. Note that the strata 1–8 (1,373 transcripts) have similar density curves but the strata 8–12 stand out as enriched in very narrowly expressed genes and having no housekeeping genes. In fact, the strata 8–12 consist of a cluster of 60 highly tissue-specific transcripts (with the mean breadth of expression of 0.06%). In contrast, 1,373 transcripts on the strata 1–7 have the breadth of expression of 0.21%. The difference between these two blocks on the X chromosome is statistically significant with *p-*value of 0.0031 (Wilcoxon rank sum test). That is to say, while all genes on the X chromosome are more tissue-specific than the autosomal average, transcripts in the strata 8–12 are exceptionally tissue-specific.
(TIF)

S3 Fig The density of transcription factor binding sites on autosomes and sex chromosomes. This figure consists of four parts identified as *a* through *d*. In part *a*, the bar chart shows the average density of transcription factor binding sites on autosomes and sex chromosomes with quality scores above certain cutoffs. The density is defined as the average number of transcription factor binding sites per one kb of DNA. The cutoffs are as follows: 0—all data, 250—data with a quality score above two hundred and fifty, 500—data with the score above five hundred, and 750—data with the score above seven hundred and fifty. The X has less than half of the density of transcription factor binding sites observed on autosomes (0.38, 0.21, 0.08, and 0.04 versus 0.93, 0.53, 0.25, and 0.13). The Y chromosome is degraded even further, with the densities of only 0.015, 0.006, 0.002, and 0.001. The label *Y-adjusted* indicates calculations performed with the length of the euchromatic Y adjusted to 20.3 Mb (to exclude masked-out PAR1 and heterochromatic Yq12). In part *b*, we find that results analogous to those for the January, 2011, data-freeze can be obtained using a broader September 2012 data-freeze [22]. In part *c*, we focus on NT2/D1 cell data only, demonstrating the same trend for the paucity of transcription factor binding sites on both sex chromosomes. In part *d*, we repeat the analysis using three transcription factors strongly expressed in testes and active in male germ-line differentiation, namely TBP, TAF1, and TAF7 [10].
(TIF)

S4 Fig The number of transcription factor binding sites on the X and the Y is lower than would be expected by the general correlation between the number of transcription factor binding sites and the gene number on the chromosome. This figure consists of eight parts identified as *a* through *h*. In parts *a*–*d*, the number of genes, the number of transcription factor binding sites, the density of genes, and the density of transcription factor binding sites (January 2011, ENCODE data-freeze) per one kb of sequence are given. In parts *e*_*–*_ *h*, scatterplots are plotted of the number of genes against the length of the chromosome (*e*), the number of genes against the number of transcription factor binding sites (*f*), the number of transcription factor binding sites against the length of the chromosome (*g*), and the density of transcription factor binding sites against the density of genes (*h*). While a strong correlation between the number of genes and the number of transcription factor binding sites (*f*), as well as the density of transcription factor binding sites and the density of genes (*h*) can be observed; chromosomes X and Y are strong outliers with the total number of transcription factor binding sites and the density of transcription factor binding sites much lower than suggested by their overall gene number and gene density.
(PDF)

S5 Fig The breadth of expression on the X chromosome. This figure consists of six parts identified as *a* through *f*. In parts *a* and *b*, scatterplots of the breadth of expression along the X chromosome are shown with an added smoothing line (*R* package *ggplot2*, method = "*loess*" parameter, span = 0.3). Chromosomal positions are shown either in base pairs (panel *a*) or as an index of the gene order (panel *b*). In parts *c*–*f*, the boxplots of the breadth of expression values in the 12 strata of the X (panel *c*), XAR versus XCR (panel *d*), XTR versus the rest (panel *e*), PAR1 versus PAR2 (panel *f*).
(JPG)

S6 Fig The breadth of expression strongly depends on promoter GC-content, but not isochore GC-content, eliminating the possibility of the impact of the different rate of biased gene conversion (bGC) on the breadth of expression on the X. This figure consists of six parts identified as *a* through *f*. Promoter regions are strongly enriched in GC-content in relation to the adjunct sequences (compare panels *a* and *d*), on both autosomes and sex chromosomes. In part *b*, a scatterplot of the breadth of expression versus promoter’s GC-content is shown (one kb window ± 500 bps from the TSS). Genes with promoter GC-content lower than 50% tend to be tissue-specific (panel *b*), but the correlation between GC-content and the breadth of expression weakens at a higher range of GC-values (i.e., more than 60%). Since the breadth of expression is not normally distributed, we applied non-parametric tests and non-linear models. Spearman correlation’s *rho* between the breadth of expression and GC-content equaled 0.1852 (*p-*value < 2.2e-16). Next, we fit a *loess* model (the red line), and draw a boxplot of residuals divided into three categories: autosomes, the X, and the Y (part *c*). Kruskall-Wallis rank sum test, a non-parametric test for heterogeneity of means across different categories, is used to find an explicit *p-*value for the difference in residuals between autosomes and the X (chi-squared = 17,059.15, degrees of freedom [*d*.*f*.] = 12,452, *p-*value < 2.2e-16). This means that the reduction in the breadth of expression on the X cannot be fully explained by the reduction in GC-content and the *p-*value lower than 2.2e-16 can be assigned directly to this effect. To compare the effect of GC-content in proximal promoters with GC-content in the surrounding sequence, we draw an analogous figure using data for the 20 kbps window around the TSS (panels *e* and *f*,*±*10 kbps from the TSS). With the exception of genes located in isochores of very low GC-content (lower than 35%), which tend to be tissue-specific, there is little correlation between isochore GC-content and the breadth of expression (panel *e*). This figure is drawn using extensively masked data (masked for assembly gaps, RepeatMasker, and Tandem Repeats Finder).
(JPG)

S7 Fig The loss of the breadth of expression on the X chromosome is much greater than suggested by its lower proximal promoter GC-content. This figure consists of five parts identified as *a* through *e*. In part *a*, we show the histogram of the means of the breadth of expression obtained from individual randomization procedures where individual genes on the X chromosome are replaced with random autosomal genes with a matched promoter GC-content. In this figure, we use 20 intervals with the breadth of 5% GC (0–5%, 5%-10%, 10%-15%, etc.) but the algorithm can work with a discretionary number of GC intervals. The observed mean breadth of expression on the X chromosome is signified with the vertical red line. In part *b*, a histogram is plotted for individual breadth of expression values from the randomization procedure broken by GC-content illustrated with color. The breadth of expression in each GC-interval is illustrated with a boxplot in part *c*. The total number of the X chromosome and autosomal genes in each GC interval is illustrated by a dot plot in part *d*. Next, we attempt to estimate what proportion of the reduction in the breadth of expression on the X chromosome could be correlated with the lower proximal promoter GC-content by preparing a *loess* model separately for autosomes and sex chromosomes (panel *e*). We find that there are an insufficient number of data points on chromosome Y to draw a reliable *loess* curve. However, a *loess* curve for the X chromosome is below that for autosomes in the entire range of promoter GC-content values.
(JPG)

S8 Fig Evidence for compensation of X-linked genes by newly spawned autosomal paralogs. This figure consists of two panels‚ one for breadth of expression (a)‚ and one for maximal expression (b). In both cases‚ the difference between autosomal paralogs of X-linked genes and autosomal-autosomal paralogs is highly statistically significant but only for duplications after the formation of X‚ and not significant for pre-existing duplications. Newly formed paralogs are defined as those mapped by phylogenetic timing to taxa Theria or younger. Pre-existing duplications are defined as those descending from duplication notes mapped by phylogenetic timing to taxa Amniota or older.
(PDF)

S9 Fig A map of the chicken synteny for the human X chromosome. This figure shows the synteny between chicken chromosomes 1 and 4 and the human chromosome X.
(PNG)

S10 Fig A correlation between tissue-specific maximal expression (TSME), binary enrichment, and binary exclusion from the X. In this figure, PEM for each brain library is calculated only in relation to non-brain libraries.
(PDF)

S1 Table The breadth of expression (BoE) and maximal expression is lower on the X in comparison to autosomes, whether calculated per transcript or per gene (either as the average or the sum). (XLSX)

S2 Table The maximal expression constraint is most likely specific to the haploid part of the X, but our conclusions are limited by the small numbers of genes in the pseudoautosomal regions. (XLSX)

S3 Table Analog enrichment (human tissues). (XLSX)

S4 Table Binary enrichment (human tissues). (XLSX)

S5 Table Binary enrichment (human primary cells). (XLSX)

S6 Table Binary enrichment (human cancer cell lines). (XLSX)

S7 Table Top maximally expressed autosomal genes. (XLSX)

S8 Table DAVID enrichment for top maximally expressed autosomal genes. (XLSX)

S9 Table DAVID enrichment for top maximally expressed X chromosome genes. (XLSX)

S10 Table Binary exclusion (human tissues). (XLSX)

S11 Table Binary exclusion (human primary cells). (XLSX)

S12 Table Binary exclusion (human cancer cell lines). (XLSX)

S13 Table The numbers of transcription factor binding sites in promoter regions (TfbsNo) on autosomes or the X depending on the analysis window size and the ENCODE quality cut-off. (XLSX)

S14 Table The mean over all averages of the breadth of expression calculated for each gene family on autosomes versus the X chromosome. (XLSX)

S15 Table The breadth of expression and maximal expression levels are lower on the X chromosome in comparison to autosomes, even after the removal of genes expressed in testis, or preferentially expressed in testis, or expressed in spermatocytes. (XLSX)

S16 Table Brain- (*B*), male- (*M*), and female-specific (*F*) expression depending on the taxon of duplication, the chromosomal location, and the retroposition status. (XLSX)

S17 Table Examples of avian brain-expressed orthologs of human brain-expressed X-linked genes. (XLSX)

S18 Table Gene families that underwent expansion on the X through tandem duplications tend to be expressed in the brain, and frequently also in reproductive tissues (either male or female). (XLSX)

S19 Table Brain tissues in the FANTOM5 tissue set (75 libraries, 27 tissues plus cerebrospinal fluid). (XLSX)

S20 Table Facultative male and female tissues in FANTOM5. (XLSX)

## Abbreviations

2ROs: 2R-ohnologs
2R-WGD: two rounds of whole genome duplication
A: autosomes
BoE: the breadth of expression
BoE_autosomal_: the mean breadth of expression on autosomes
BoE_X_: the mean breadth of expression on the X
BED: Browser Extensible Data
BEX1: brain expressed, X-linked 1
bps: base pairs
CAGE: Cap Analysis of Gene Expression
d.f.: degrees of freedom
ENCODE: The Encyclopedia of DNA Elements
FANTOM5: Functional Annotation of the Mammalian Genome
GC3: GC-content at third sites
kb: kilobase
kbps: kilo base pairs
PAR1: pseudoautosomal region
SD: standard deviation
TfbsNo: number of transcription factor binding sites per promoter
TPM: tags per million
TSME: tissue-specific maximal expression
TSSes: transcriptional start sites
MAGE: melanoma antigen E tumor-specific antigens
NCBI: National Centre for Biotechnology Information
RPKM: reads per kilobase per million mapped reads
X: the X chromosome
