# Supplementary material for: The Constrained Maximal Expression Level Owing to Haploidy Shapes Gene Content on the Mammalian X Chromosome
Source: PLoS Biol. 2015 Dec 18;13(12):e1002315. doi: 10.1371/journal.pbio.1002315 (PMC4686125; doi:10.1371/journal.pbio.1002315)
Supplement: S1 Fig — This figure consists of three panels marked as a–c. Data for human tissues (a), primary cells (b), and cancer cell-lines (c) are shown. Values above one on the x-axis signify exclusion from the X. The greater the degree of the exclusion, the greater the value on the x-axis. These charts are independent of the strength of expression of individual genes, as all data points were first converted into a binary (“on” or “off”). It is striking that gene expression in all samples is under-represented on the X by this measure (although brain tissues are least excluded). Details can be found in S10–S12 Tables. (PDF) [file pbio.1002315.s001.pdf]

(a) human tissues

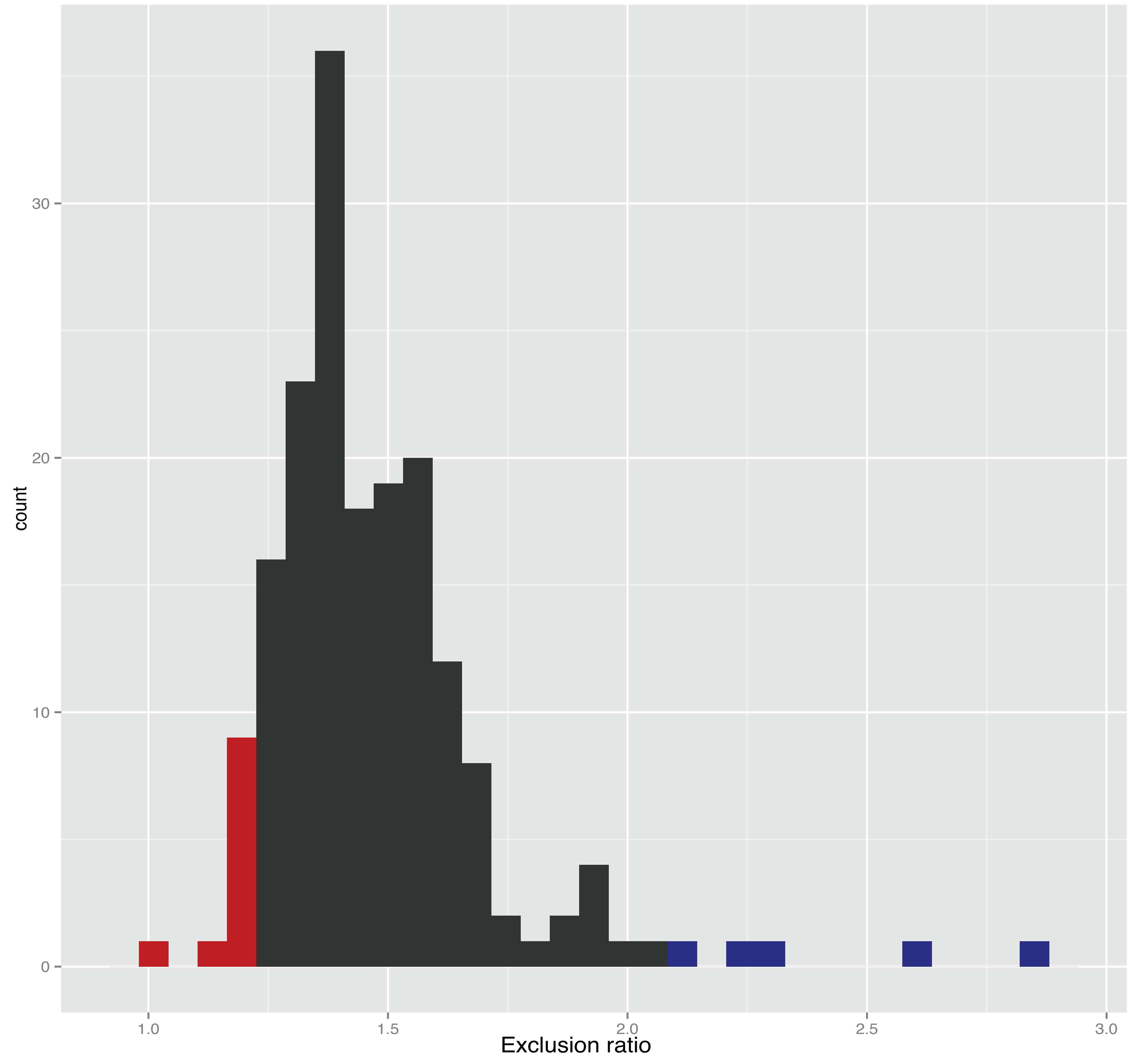

Sample	*exclusion ratio*
substantia nigra adult	1.04
putamen adult	1.2
substantia nigra adult	1.18
medial temporal gyrus adult	1.19
parietal cortex adult	1.2
(...)	
pancreas adult	2.1
skin palm	2.2
achilles tendon	2.3
eye muscle inferior rectus	2.6
tongue epidermis	2.9

(b) human primary cells

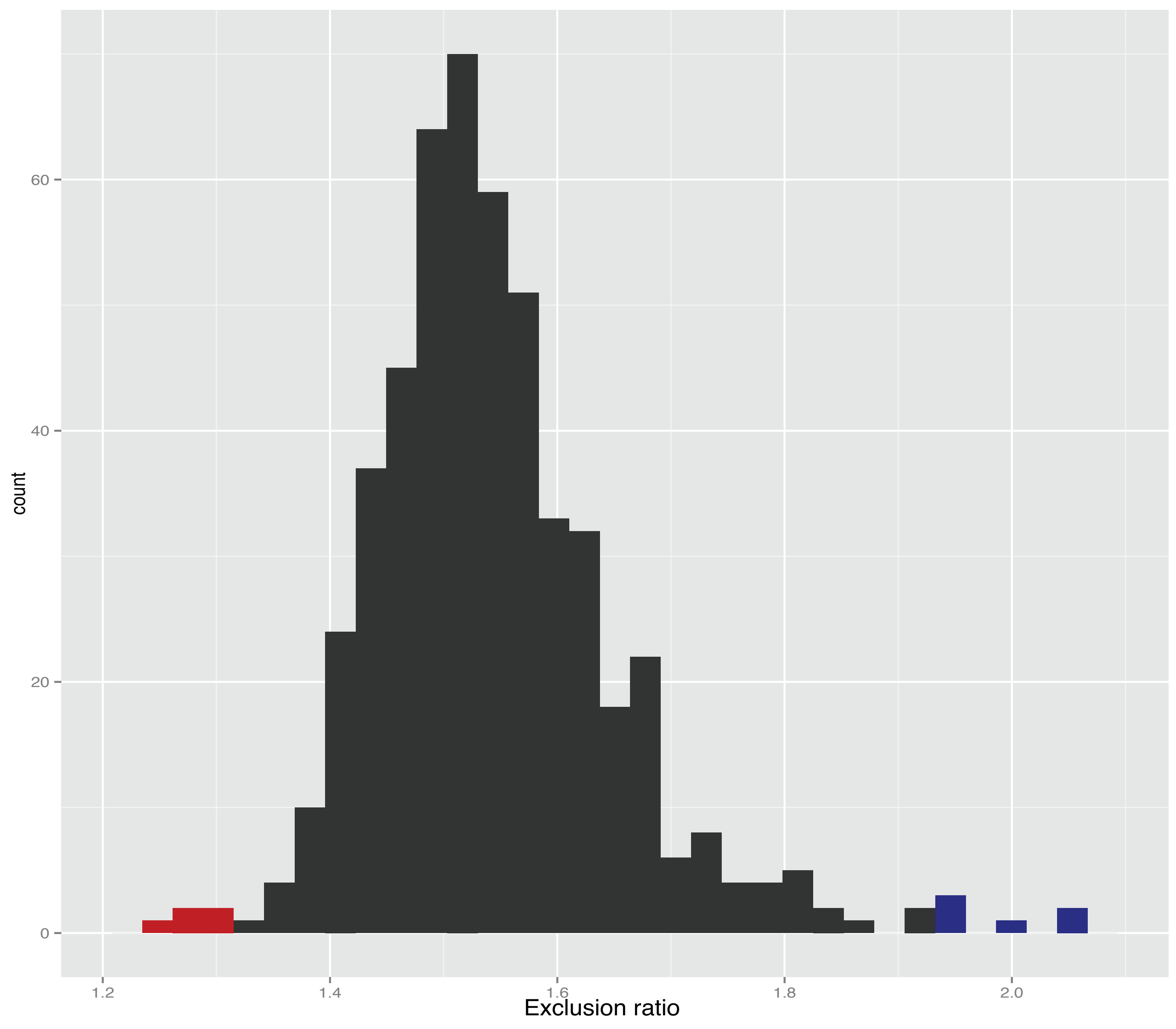

Sample	*exclusion ratio*
Mast cell	1.3
Neural stem cells	1.3
Lens Epithelial Cells	1.3
Mast cell donor	1.3
Adipocyte subcutaneous	1.3
(...)	
Mesenchymal Stem Cells	1.9
Tracheal Epithelial Cells	1.9
Bronchial	1.9
Alveolar Epithelial cells	2
Hepatocyte	2.1

(c) human cancer cell lines

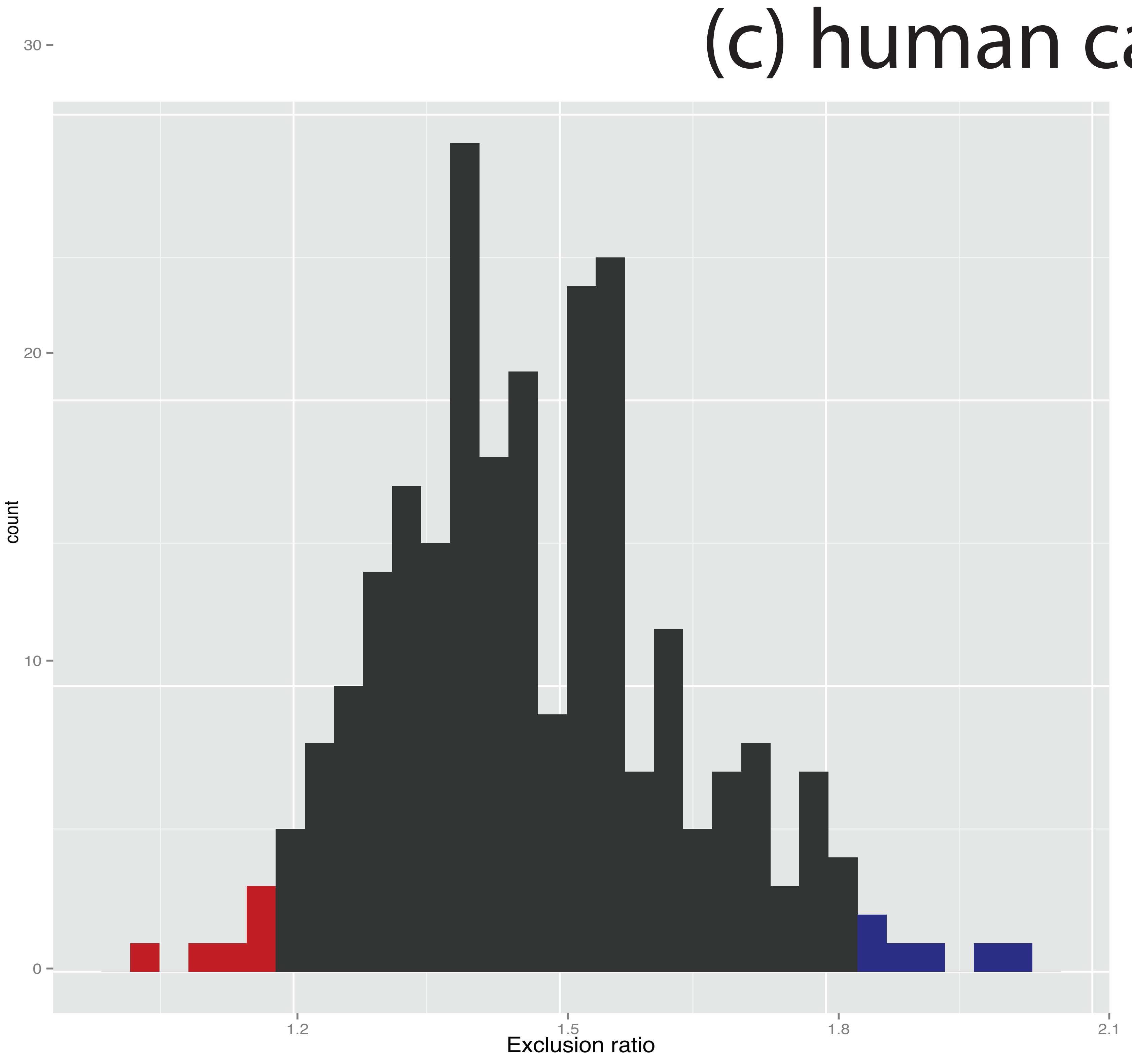

Sample	*exclusion ratio*
teratocarcinoma cell line	1
colon carcinoma cell line	1
large cell lung carcinoma	1.1
synovial sarcoma cells	1.2
neuroectodermal tumor	1.2
(...)	
acute myeloid leukemia	1.9
chronic lymphocytic leukemia	1.9
prostate cancer cells	1.9
oral squamous carcinoma	1.9
bile duct carcinoma	2
