# Supplementary figures and images for: The Constrained Maximal Expression Level Owing to Haploidy Shapes Gene Content on the Mammalian X Chromosome
Source: PLoS Biol. 2015 Dec 18;13(12):e1002315. doi: 10.1371/journal.pbio.1002315 (PMC4686125; doi:10.1371/journal.pbio.1002315)

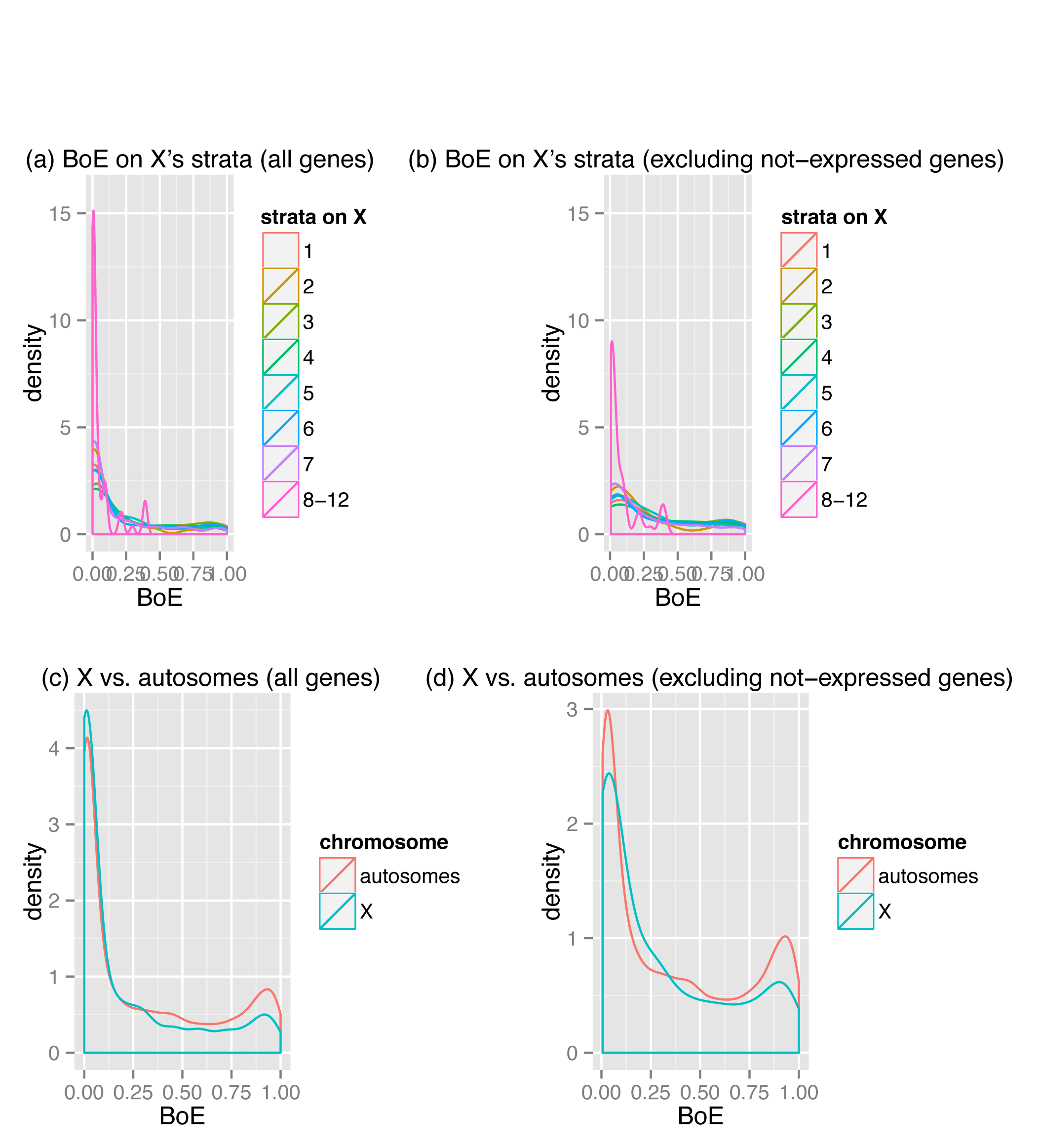

Supplement: S2 Fig — This figure consists of four panels marked as a through d and showing density plots for the breadth of expression. Either all genes (a and c) or only-expressed genes are shown (b and d). In panels c and d, the breadth of expression is compared between autosomes and the entire X chromosome, while in panels a and b the breadth of expression is divided with respect to X’s strata. Note that the strata 1–8 (1,373 transcripts) have similar density curves but the strata 8–12 stand out as enriched in very narrowly expressed genes and having no housekeeping genes. In fact, the strata 8–12 consist of a cluster of 60 highly tissue-specific transcripts (with the mean breadth of expression of 0.06%). In contrast, 1,373 transcripts on the strata 1–7 have the breadth of expression of 0.21%. The difference between these two blocks on the X chromosome is statistically significant with p-value of 0.0031 (Wilcoxon rank sum test). That is to say, while all genes on the X chromosome are more tissue-specific than the autosomal average, transcripts in the strata 8–12 are exceptionally tissue-specific. (TIF) [file pbio.1002315.s002.tif]

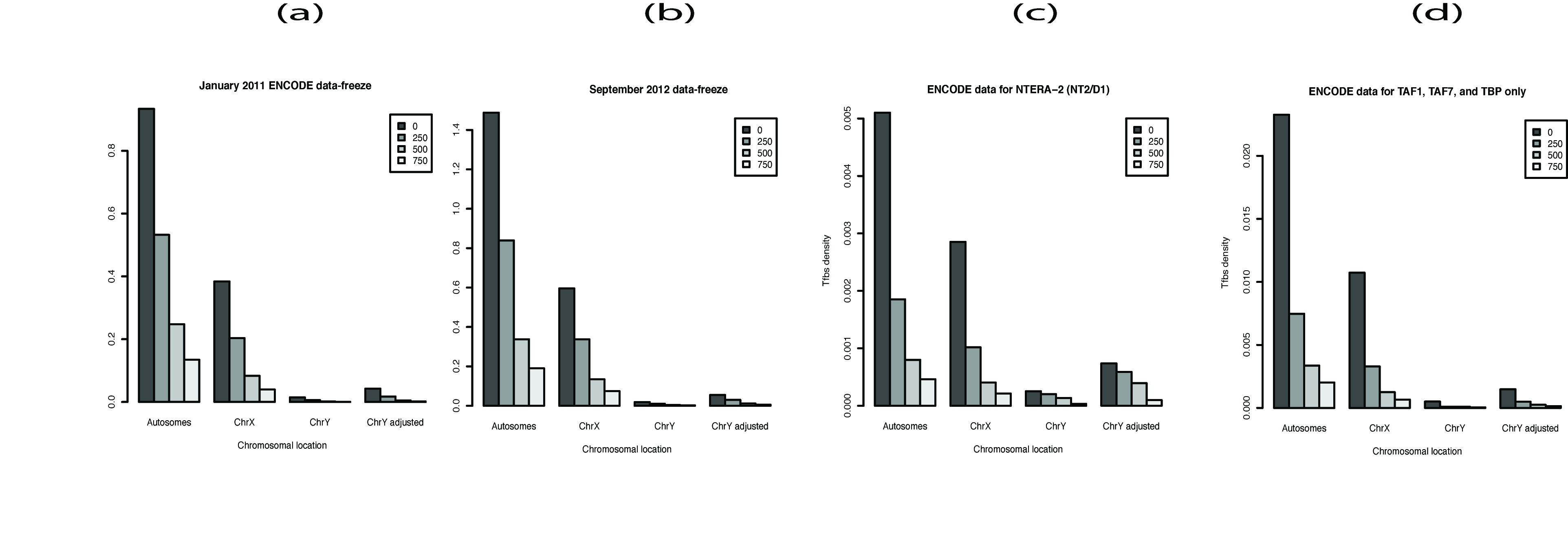

Supplement: S3 Fig — This figure consists of four parts identified as a through d. In part a, the bar chart shows the average density of transcription factor binding sites on autosomes and sex chromosomes with quality scores above certain cutoffs. The density is defined as the average number of transcription factor binding sites per one kb of DNA. The cutoffs are as follows: 0—all data, 250—data with a quality score above two hundred and fifty, 500—data with the score above five hundred, and 750—data with the score above seven hundred and fifty. The X has less than half of the density of transcription factor binding sites observed on autosomes (0.38, 0.21, 0.08, and 0.04 versus 0.93, 0.53, 0.25, and 0.13). The Y chromosome is degraded even further, with the densities of only 0.015, 0.006, 0.002, and 0.001. The label Y-adjusted indicates calculations performed with the length of the euchromatic Y adjusted to 20.3 Mb (to exclude masked-out PAR1 and heterochromatic Yq12). In part b, we find that results analogous to those for the January, 2011, data-freeze can be obtained using a broader September 2012 data-freeze [22]. In part c, we focus on NT2/D1 cell data only, demonstrating the same trend for the paucity of transcription factor binding sites on both sex chromosomes. In part d, we repeat the analysis using three transcription factors strongly expressed in testes and active in male germ-line differentiation, namely TBP, TAF1, and TAF7 [10]. (TIF) [file pbio.1002315.s003.tif]

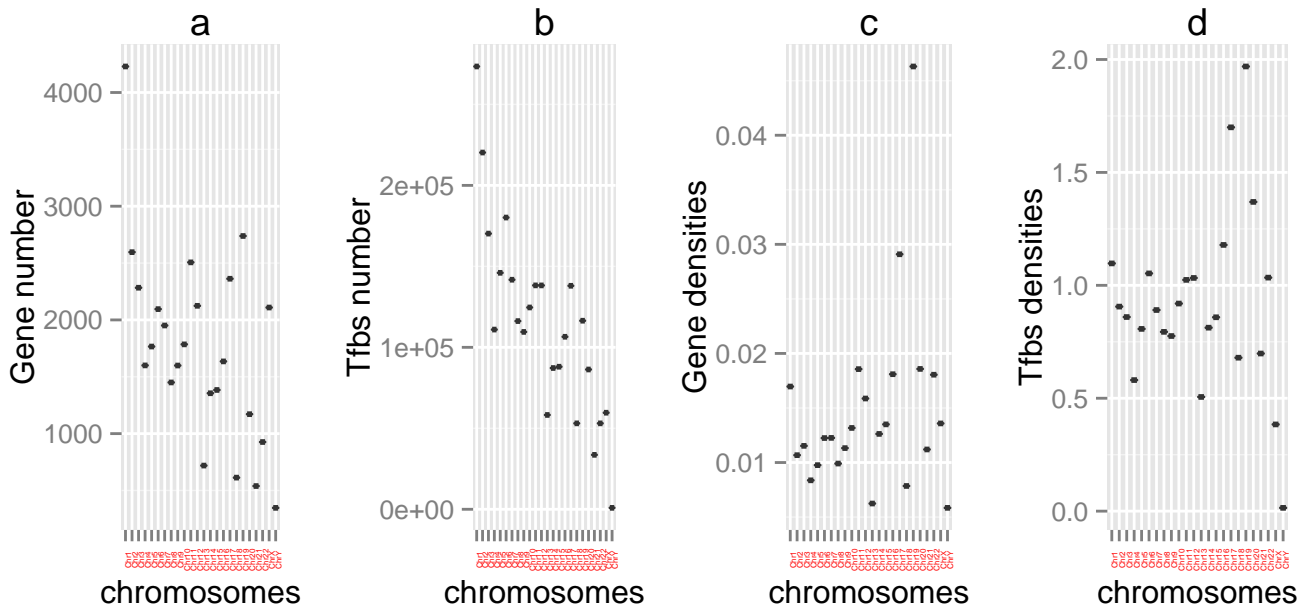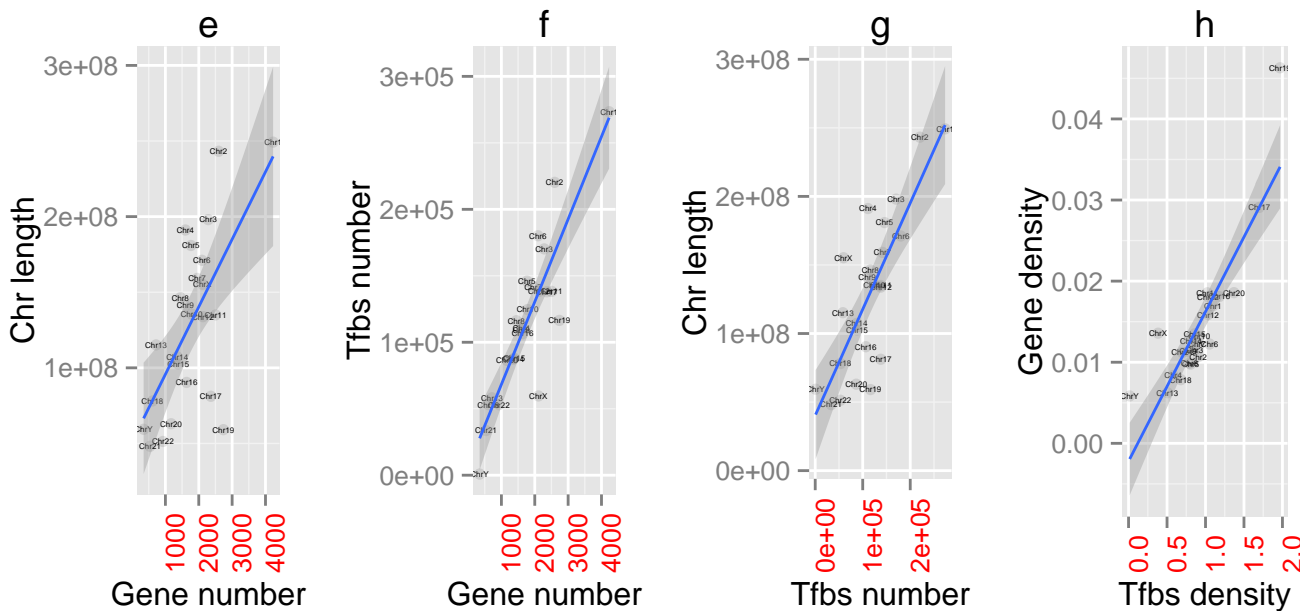

Supplement: S4 Fig — This figure consists of eight parts identified as a through h. In parts a–d, the number of genes, the number of transcription factor binding sites, the density of genes, and the density of transcription factor binding sites (January 2011, ENCODE data-freeze) per one kb of sequence are given. In parts e – h, scatterplots are plotted of the number of genes against the length of the chromosome (e), the number of genes against the number of transcription factor binding sites (f), the number of transcription factor binding sites against the length of the chromosome (g), and the density of transcription factor binding sites against the density of genes (h). While a strong correlation between the number of genes and the number of transcription factor binding sites (f), as well as the density of transcription factor binding sites and the density of genes (h) can be observed; chromosomes X and Y are strong outliers with the total number of transcription factor binding sites and the density of transcription factor binding sites much lower than suggested by their overall gene number and gene density. (PDF) [file pbio.1002315.s004.pdf]

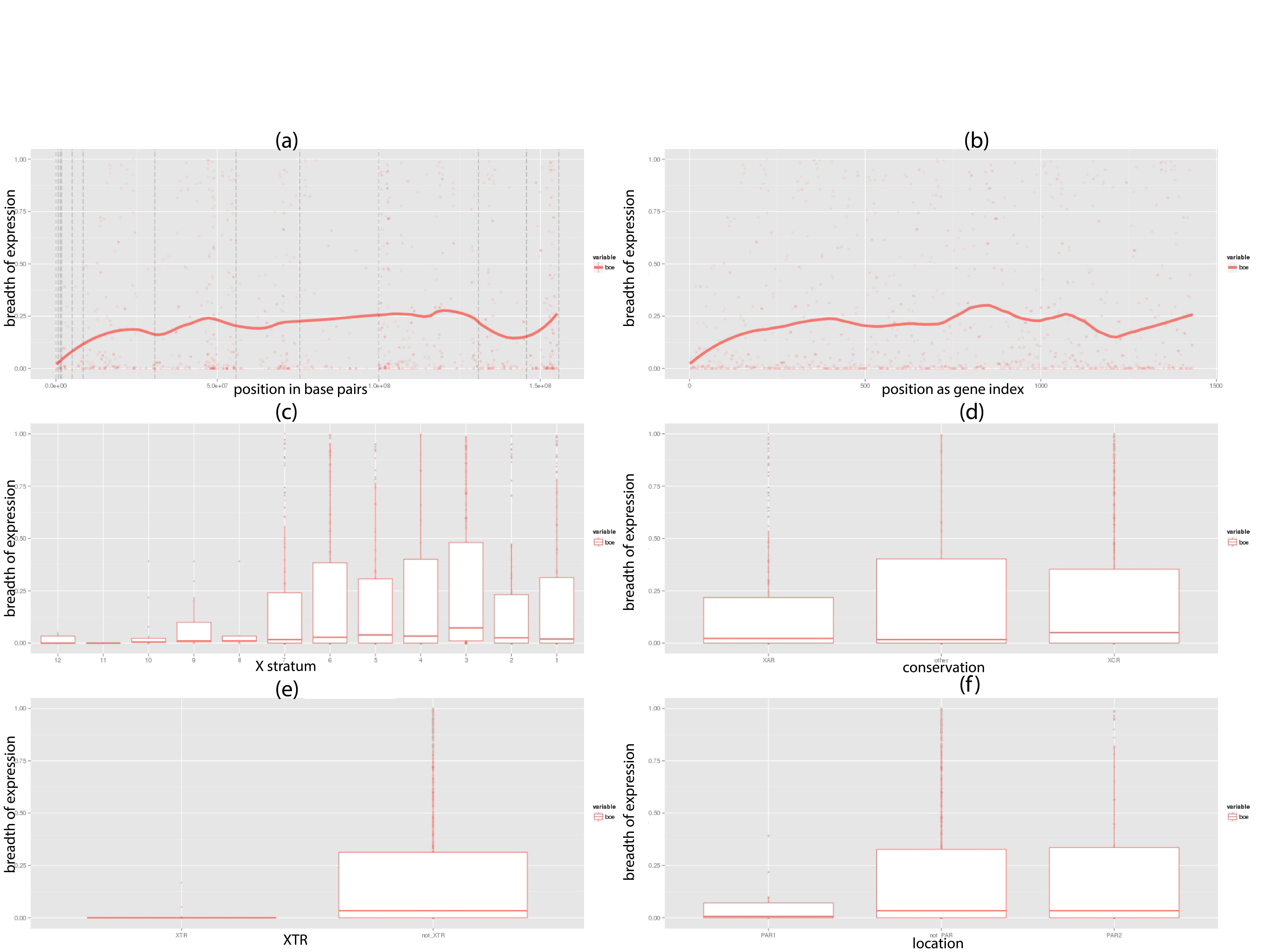

Supplement: S5 Fig — This figure consists of six parts identified as a through f. In parts a and b, scatterplots of the breadth of expression along the X chromosome are shown with an added smoothing line (R package ggplot2, method = "loess" parameter, span = 0.3). Chromosomal positions are shown either in base pairs (panel a) or as an index of the gene order (panel b). In parts c–f, the boxplots of the breadth of expression values in the 12 strata of the X (panel c), XAR versus XCR (panel d), XTR versus the rest (panel e), PAR1 versus PAR2 (panel f). (JPG) [file pbio.1002315.s005.jpg]

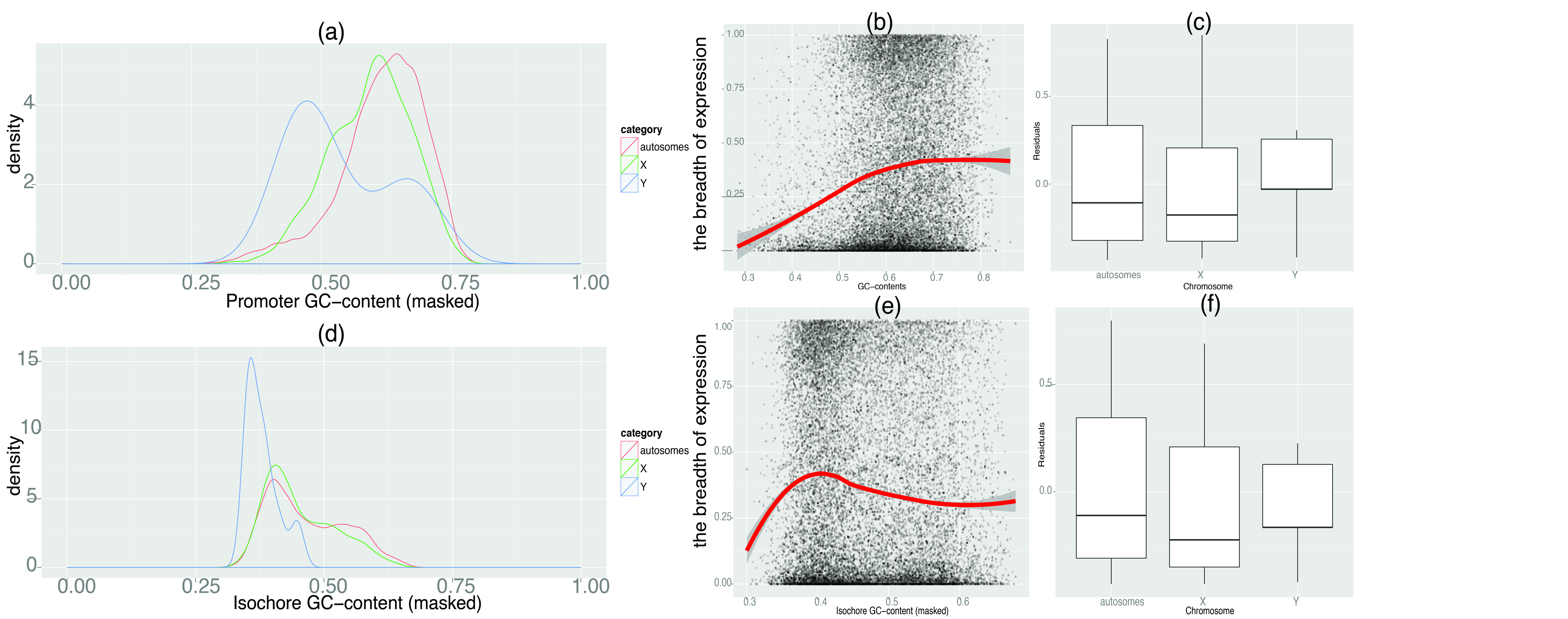

Supplement: S6 Fig — This figure consists of six parts identified as a through f. Promoter regions are strongly enriched in GC-content in relation to the adjunct sequences (compare panels a and d), on both autosomes and sex chromosomes. In part b, a scatterplot of the breadth of expression versus promoter’s GC-content is shown (one kb window ± 500 bps from the TSS). Genes with promoter GC-content lower than 50% tend to be tissue-specific (panel b), but the correlation between GC-content and the breadth of expression weakens at a higher range of GC-values (i.e., more than 60%). Since the breadth of expression is not normally distributed, we applied non-parametric tests and non-linear models. Spearman correlation’s rho between the breadth of expression and GC-content equaled 0.1852 (p-value < 2.2e-16). Next, we fit a loess model (the red line), and draw a boxplot of residuals divided into three categories: autosomes, the X, and the Y (part c). Kruskall-Wallis rank sum test, a non-parametric test for heterogeneity of means across different categories, is used to find an explicit p-value for the difference in residuals between autosomes and the X (chi-squared = 17,059.15, degrees of freedom [d.f.] = 12,452, p-value < 2.2e-16). This means that the reduction in the breadth of expression on the X cannot be fully explained by the reduction in GC-content and the p-value lower than 2.2e-16 can be assigned directly to this effect. To compare the effect of GC-content in proximal promoters with GC-content in the surrounding sequence, we draw an analogous figure using data for the 20 kbps window around the TSS (panels e and f,±10 kbps from the TSS). With the exception of genes located in isochores of very low GC-content (lower than 35%), which tend to be tissue-specific, there is little correlation between isochore GC-content and the breadth of expression (panel e). This figure is drawn using extensively masked data (masked for assembly gaps, RepeatMasker, and Tandem Repeats Finder). (JPG) [file pbio.1002315.s006.jpg]

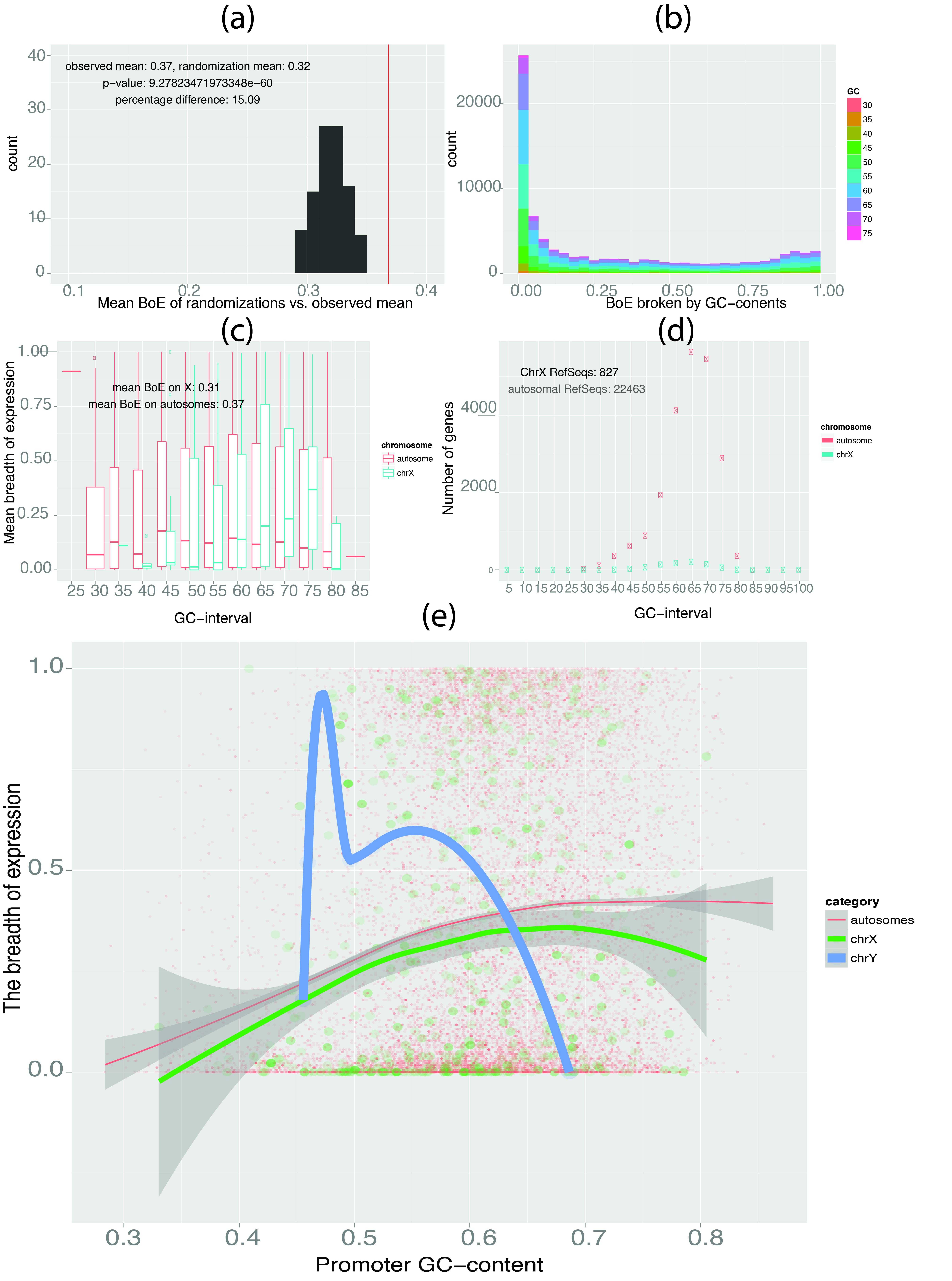

Supplement: S7 Fig — This figure consists of five parts identified as a through e. In part a, we show the histogram of the means of the breadth of expression obtained from individual randomization procedures where individual genes on the X chromosome are replaced with random autosomal genes with a matched promoter GC-content. In this figure, we use 20 intervals with the breadth of 5% GC (0–5%, 5%-10%, 10%-15%, etc.) but the algorithm can work with a discretionary number of GC intervals. The observed mean breadth of expression on the X chromosome is signified with the vertical red line. In part b, a histogram is plotted for individual breadth of expression values from the randomization procedure broken by GC-content illustrated with color. The breadth of expression in each GC-interval is illustrated with a boxplot in part c. The total number of the X chromosome and autosomal genes in each GC interval is illustrated by a dot plot in part d. Next, we attempt to estimate what proportion of the reduction in the breadth of expression on the X chromosome could be correlated with the lower proximal promoter GC-content by preparing a loess model separately for autosomes and sex chromosomes (panel e). We find that there are an insufficient number of data points on chromosome Y to draw a reliable loess curve. However, a loess curve for the X chromosome is below that for autosomes in the entire range of promoter GC-content values. (JPG) [file pbio.1002315.s007.jpg]

(a)

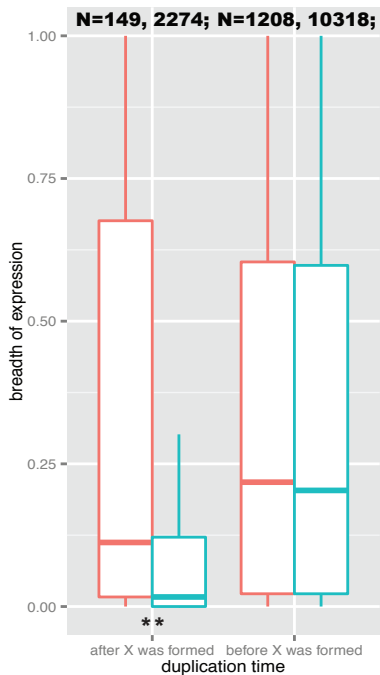

(b)

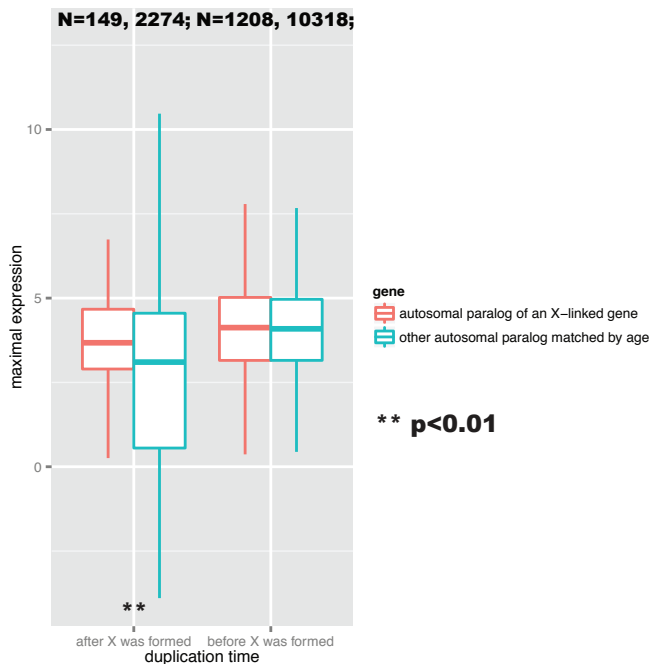

Supplement: S8 Fig — This figure consists of two panels‚ one for breadth of expression (a)‚ and one for maximal expression (b). In both cases‚ the difference between autosomal paralogs of X-linked genes and autosomal-autosomal paralogs is highly statistically significant but only for duplications after the formation of X‚ and not significant for pre-existing duplications. Newly formed paralogs are defined as those mapped by phylogenetic timing to taxa Theria or younger. Pre-existing duplications are defined as those descending from duplication notes mapped by phylogenetic timing to taxa Amniota or older. (PDF) [file pbio.1002315.s008.pdf]

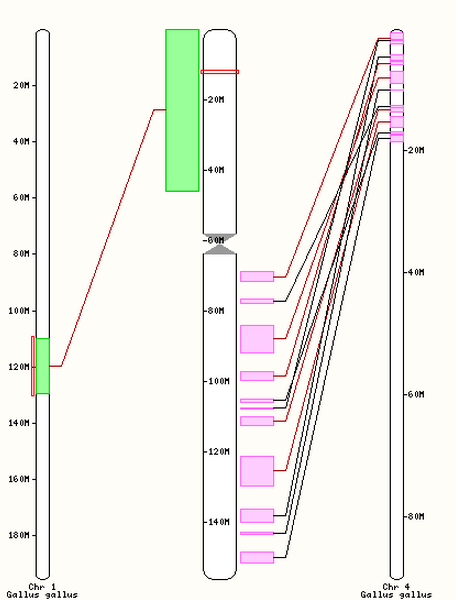

Supplement: S9 Fig — This figure shows the synteny between chicken chromosomes 1 and 4 and the human chromosome X. (PNG) [file pbio.1002315.s009.png]

p-value: 0.0321993444; rho: -0.1615

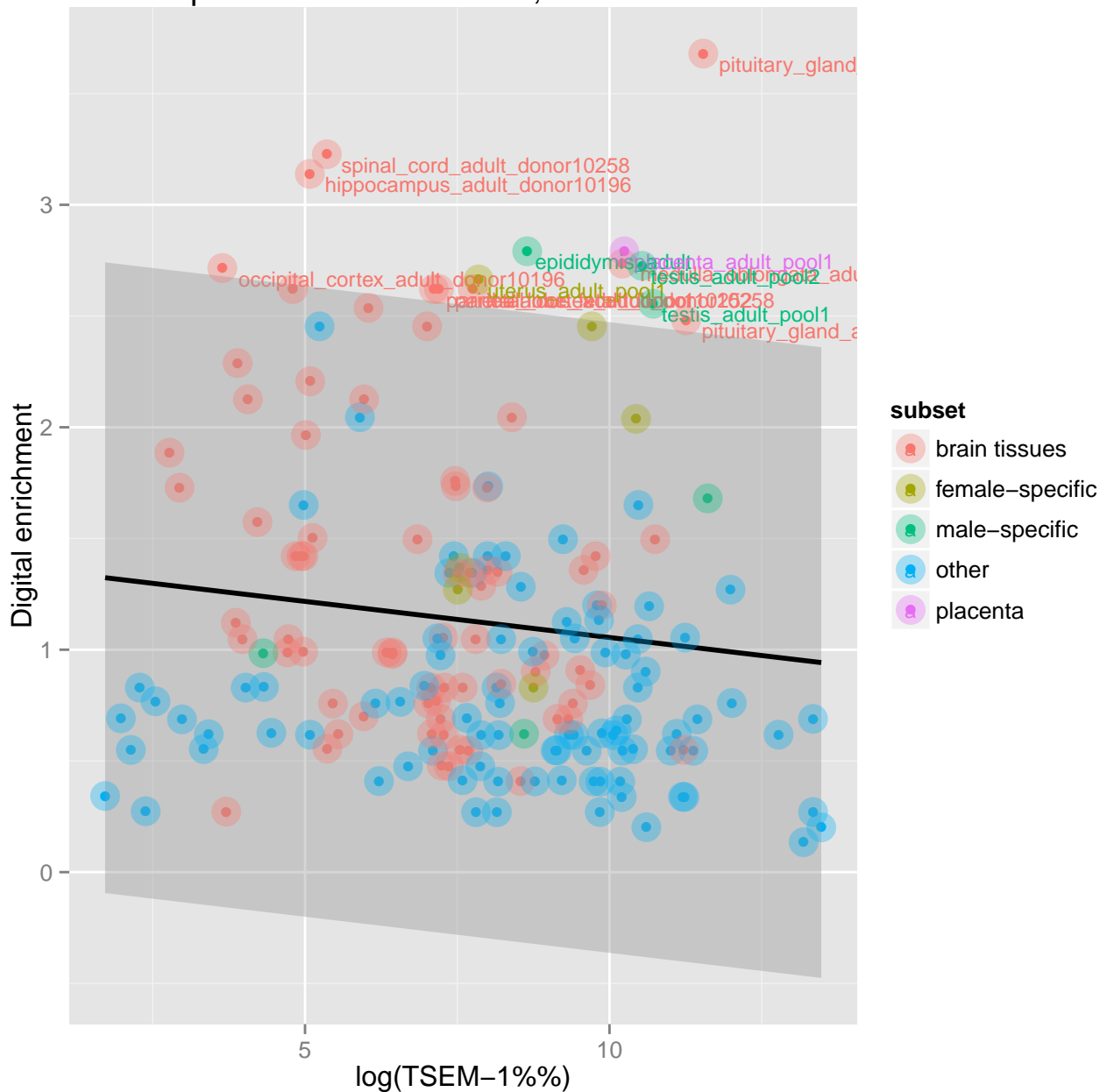

Supplement: S10 Fig — In this figure, PEM for each brain library is calculated only in relation to non-brain libraries. (PDF) [file pbio.1002315.s010.pdf]
